# The Effects of Forest Harvesting on Total and Methylmercury Concentrations in Surface Waters Depend on Harvest Practices and Physical Site Characteristics

**DOI:** 10.1021/acs.est.5c02787

**Published:** 2025-07-22

**Authors:** Karin Eklöf, Heleen de Wit, Chris S. Eckley, Collin A. Eagles-Smith, Susan L. Eggert, Robert W. Mackereth, Ulf Skyllberg, Liisa Ukonmaanaho, Matti Verta, Craig Allan, Erik J. S. Emilson, Karen A. Kidd, Carl P. J. Mitchell, John Munthe, Tapani Sallantaus, Joel Segersten, Andrea G. Bravo, Randall K. Kolka, Colin P. R. McCarter, Petri Porvari, Eva Ring, Stephen D. Sebestyen, Ulf Sikström, Therese Sahlén Zetterberg

**Affiliations:** † Department of Aquatic Sciences and Assessment, 8095 Swedish University of Agricultural Sciences, Uppsala SE-75007, Sweden; ‡ Norwegian Institute for Water Research, Økernveien 94, Oslo 0579, Norway; § Center of Biogeochemistry in the Anthropocene, Department of Biosciences, University of Oslo, P.O. Box 1066, Oslo 0371, Norway; ∥ United States Environmental Protection Agency, Region 10, 1200 Sixth Avenue, Seattle, Washington 98101, United States; ⊥ Forest and Rangeland Ecosystem Science Center, United States Geological Survey, 3200 SW Jefferson Way, Corvallis, Oregon 97331, United States; # 98654 United States Department of Agriculture Forest Service, Northern Research Station, Grand Rapids, Minnesota 55744, United States; ¶ Centre for Northern Forest Ecosystem Research, Ontario Ministry of Natural Resources, 421 James St S., Thunder Bay,Ontario, P7E 2 V6, Canada; ∇ Department of Forest Ecology and Management, Swedish University of Agricultural Sciences, Umeå SE-90183, Sweden; ○ 419837 Natural Resources Institute Finland (LUKE), Latokartanonkaari 9, Helsinki 00790, Finland; ⧫ 73144 Finnish Environment Institute (Syke), Latokartanonkaari 11, Helsinki 00790, Finland; †† Department of Earth, Environmental and Geographical Sciences, University of North Carolina Charlotte, Charlotte, North Carolina 28223, United States; ‡‡ 6314 Natural Resources Canada, Great Lakes Forestry Centre, 1219 Queen St. E, Sault Ste. Marie, Ontario P6A2E3, Canada; §§ Department of Biology, 3710 McMaster University, Hamilton, Ontario L8S4K1, Canada; ∥∥ Department of Physical and Environmental Sciences, 33530 University of Toronto Scarborough, Toronto, Ontario MIC1A4, Canada; ⊥⊥ IVL Swedish Environmental Research Institute, P.O. Box 530 21, Göteborg SE-40014, Sweden; ## Departament de Biologia Marina i Oceanografia, 58341 Institut de Ciències del Mar, ICM-CSIC, Barcelona, Catalunya 08003, Spain; ¶¶ Department of Biology, Chemistry, and Geography, 226783 Nipissing University, North Bay, Ontario P1B8L7, Canada; ∇∇ The Forestry Research Institute of Sweden, 8098 Skogforsk, Uppsala Science Park, Uppsala SE-75183, Sweden; ○○ Department of Soil and Environment, Swedish University of Agricultural Sciences, Uppsala SE-75007, Sweden

**Keywords:** forestry, clear-cut, methylmercury, soil disturbance, mitigation methods

## Abstract

Forest harvesting can lead to mercury (Hg) mobilization from soils to aquatic habitats and promote the transformation of inorganic Hg to highly neurotoxic and bioaccumulative methyl-Hg (MeHg). Multiple past studies reveal broad variation of stream water MeHg and total Hg (THg) concentration responses to forest harvesting, which has confounded messaging to forest and resource managers. To advance beyond divergent and sometimes contradictory findings, we synthesized information for 23 previously studied catchments in North America and Fennoscandia and compiled a uniform set of soil, landscape, and harvesting properties to identify forest management, riparian, and hillslope factors that influence responses of stream water MeHg and THg concentrations. From this synthesis, we found catchments with high soil moisture and organic soil layers >100 cm to be at highest risk for disturbance-induced increases in MeHg formation after harvest but not necessarily affecting concentrations of MeHg in stream waters. Instead, the combination of MeHg formation in soils along with factors that affect mobilization with runoff to streams most influenced how forest harvest affects MeHg concentrations in stream waters.

## Graphical abstract

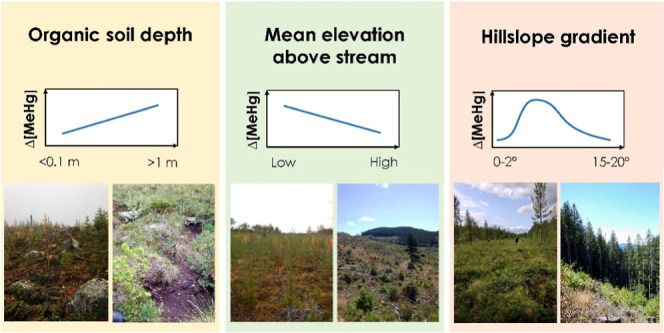

## 1 Introduction

Forest soils are important reservoirs of mercury (Hg) originating from long-range transboundary air pollution and historic point sources.^1^ Over 90% of atmospherically deposited Hg in boreal forest catchments is retained in soils.^2^ The Hg accumulated in soils slowly leaches into groundwater and runoff. The transformation of inorganic Hg to the highly neurotoxic and bioaccumulative methyl-Hg (MeHg) is stimulated at aerobic–anaerobic interfaces in water-saturated soils or in the water column where Hg-methylating microorganisms, such as sulfate-reducing bacteria,^3,4^ iron-reducing bacteria,^5,6^ methanogens,^7^ or syntrophs,^8^ exist and thrive. The mobilization and methylation of Hg are contributing to the loading and biomagnification of Hg downstream.^9^ Due to the biomagnification of Hg within aquatic food webs, the total Hg content in inland fish commonly exceeds levels that the World Health Organization (WHO) deems potentially harmful for human consumption (>0.5 mg kg^–1^) across many water bodies in, e.g., Finland, Sweden, and Norway,^10,11^ as well as in North America.^12^

Forest harvest may enhance Hg mobilization from soil and promote the formation of MeHg.^9^ Tree removal during forest harvest lowers transpiration and may also increase snow cover, both of which cause elevated groundwater levels, increased soil moisture, and possibly overland flow.^13^ Higher groundwater levels can change the redox status of the soil and thereby promote Hg-methylation.^14–16^ More near-surface flow paths can also facilitate mobilization of inorganic Hg and MeHg from soils to surface waters.^17,18^ Heavy machinery movement during forestry operations, such as skidder and forwarder traffic, increases the risk of overland flow due to soil compaction, and formation of wheel ruts could increase the erosion of soil particles and associated Hg.^19^ Water-filled wheel ruts can increase the risk of MeHg formation. Harvesting in areas with a low elevation above stream level may further increase the risk of developing waterlogged anoxic environments. Post-harvest mechanical site preparation or stump harvest may further increase the risk of elevated MeHg formation. Mechanical site preparation exposes the mineral soil and forms mounds or tilts where the new seeds or seedlings can be planted.^20^ Stump removal can disrupt the soil structure, further increasing the risk of water-filled cavities with high MeHg formation potential.^21^ The degree of soil disturbance—and likely MeHg formation and mobilization—caused by these management practices is also driven by soil properties, season and weather conditions when operations are carried out, topography, tree volume to be extracted, and onsite practices.^22^ The soil bearing capacity, i.e., soil susceptibility to compaction, may be lower in areas of low stone and boulder content,^23^ high soil moisture, and low bulk density such as peat soils.^24^ In steep slopes and well-drained soils, anoxic conditions may not develop after harvest,^25^ but erosion of soil-bound Hg can instead be high. The composition of organic matter has been found to influence both MeHg mobilization^26^ and formation^27^ in surface waters. The soil nutrient content has also been shown to influence net MeHg formation in wetlands.^28^

Studies evaluating forest harvesting responses reveal great variation in Hg mobilization, especially regarding MeHg concentrations in stream waters—from minimal^e.g.^ ^29–32^ to intermediate (up to 100%^e.g.^ ^20,33^ ) up to many-fold concentration increases.^34–37^ Others found forest harvesting effects on only total Hg (THg) or dissolved Hg (DHg) concentrations^25^ or on both the THg and MeHg fluxes in runoff water.^38^ Downstream of forest harvest, increased Hg in fish,^39^ invertebrates,^40^ zooplankton,^41^ periphyton,^42^ and wildlife^40^ has been reported. The variation in impact among sites and species are, however, large.^39,41–45^

Even though considerable progress has been made in defining fundamental forest harvesting effects on THg and MeHg concentrations in stream water, knowledge about site-specific factors and mitigation measures that account for this variation is limited. Hence, it is difficult to inform forest managers on measures that could mitigate the impact of forest harvest on Hg-methylation and MeHg in runoff water. In this study, we collected soil samples and other site-specific data and conducted geographical information systems (GIS) analysis to address disparate results of previously published studies of post-harvest MeHg and THg concentrations in stream water. We studied 23 catchments in Fennoscandia and North America that examined post-harvest impacts on Hg (Supporting Information, Table S1). The data were collected to identify and where possible quantify factors influencing the site-specific variation in effects of forest harvest with regard to Hg mobilization and methylation to improve the scientific basis for possible measures to prevent increases of THg and MeHg concentrations in surface water after harvest. We aimed to determine whether aqueous MeHg concentrations increase after harvest with (1) increasing harvested proportion of the catchment; (2) decreasing mean elevation above the stream of the harvested area; (3) increasing thickness of the soil organic layer; (4) decreasing stone and boulder content in soils; (5) decreasing hillslope gradients; (6) decreasing carbon (C) to nitrogen (N) ratio of soils, as a potential proxy for higher tree growth, evapotranspiration, and organic matter quality; (7) increased rutting of soils; and (8) lack of riparian buffer along the stream.

In addition, we also aimed to determine if THg concentrations in stream waters increased after harvest following factors 1–4 and 6–8, and if post-harvesting THg concentrations in stream waters increased with increasing hillslope gradient.

## 2 Methods

### 2.1 Site Description and Experimental Design

We focused on catchments with previous studies of Hg in stream and ground/soil waters following forest harvesting with or without subsequent mechanical site preparation and/or stump harvest. Forest harvesting in this study refers to clear-cutting (*n* = 21) and selective cutting (*n* = 2; Supporting Information, Table S1). The 23 catchments included in this study originated from 13 published studies^14,18,20,21,25,29–32,34,35,46^ and 1 publication in preparation.^47^ Both natural and artificial (ditches and straightened channels) water courses were sampled in the original studies. Hereafter, we use “streams” both for natural streams and artificial ditches. The catchments are distributed from Oregon, USA, in the west to Finland in the east, with sites in the USA, Canada, Norway, Sweden, and Finland (Figure 1). We restricted this study to include (i) catchments from the boreal and hemi boreal vegetation zones in the northern hemisphere, (ii) studies where effects of forest harvest included THg and/or MeHg measurement in surface or ground/soil water, (iii) studies where the THg and/or MeHg concentrations from the harvested area were compared with those from a nearby reference area (without harvest) and/or a reference period (before harvest), and (iv) studies where time series of Hg concentrations in stream waters (or ground/soil waters) from repeated sampling were available.

**Figure 1:**
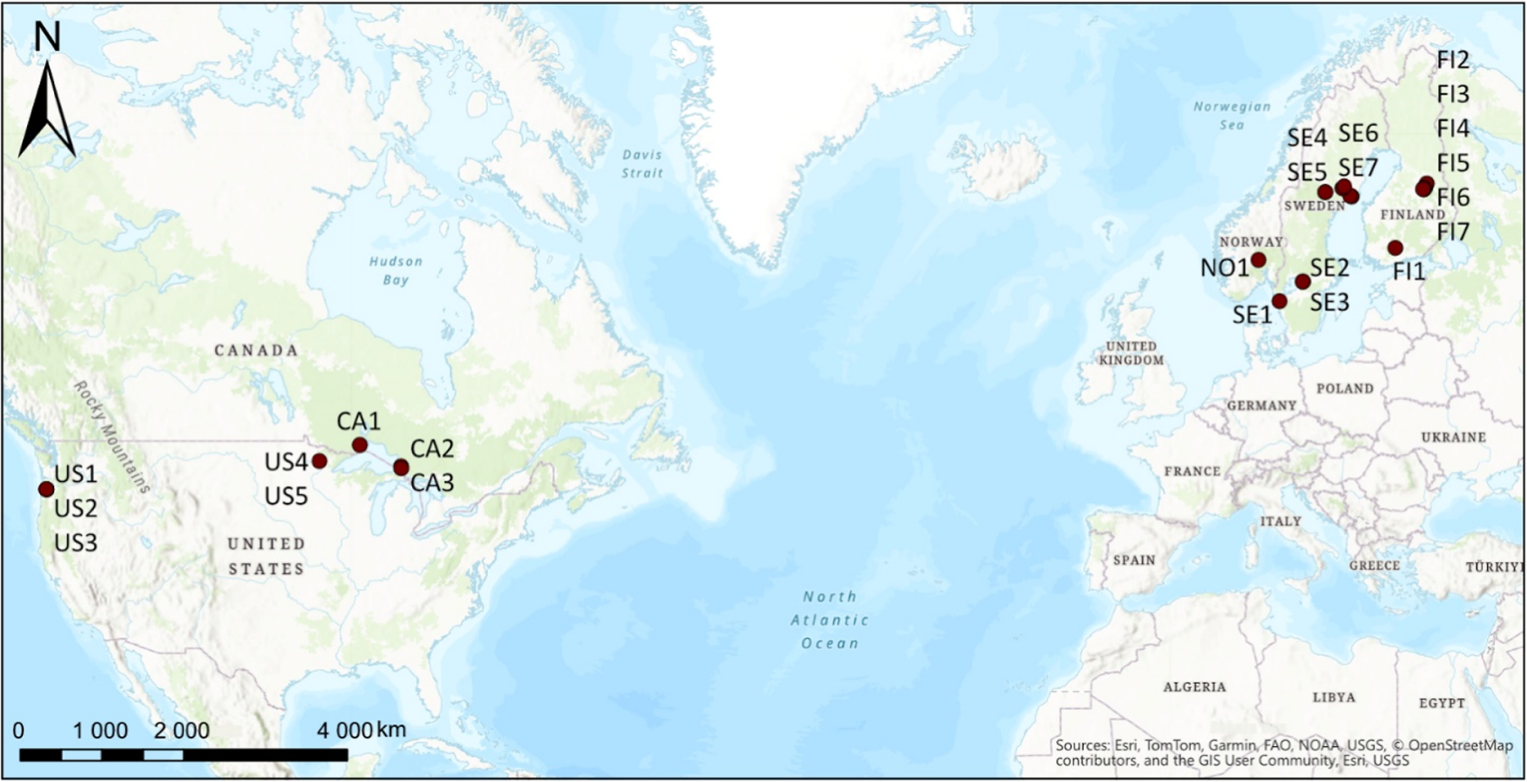
Location of the 23 study catchments in the USA, Canada, Norway, Sweden, and Finland. Due to limited space, the country code and a random number, but not the treatment code, are shown in the figure. Background map image (World Topographic Map) is the intellectual property of Esri and is used herein under license. Copyright © 2025 Esri and its licensors. All rights reserved.

All 15 catchments in Fennoscandia were covered by boreal coniferous forest. Of the catchments in North America, four had deciduous forest, three had coniferous, and one had mixed forest (Supporting Information, Table S1). The site names reflect the country code and random number, followed by the treatment code for clear-cutting (Cc), selective cutting (Sc), mechanical site preparation (Sp), stump harvest (Sh), logging residue harvest (Rh), and logging trail (Lt). In two of the Canadian catchments, the forest harvest followed a selective cutting protocol (CA2-Sc and CA3-Sc). In seven of the catchments (US1-Cc, US2-Cc, US3-Cc, US4-Cc, CA1-Cc, NO1-Cc, SE6-Cc, and SE6-Cc), clear-cutting only was evaluated, and in one catchment, the logging residues were removed after clear-cutting (US5-CcRh). Clear-cutting was followed by site preparation in six catchments (SE2-CcSp, SE4-CcSp, SE5-CcSp, FI1-CcSp, FI3-CcSp, and FI4-CcSp) and stump harvest in one catchment (SE3-CcSh). In FI2-CcSpSh, clear-cutting was followed by site preparation and stump harvest, and these treatments were also followed by logging residue harvest in sites FI5-CcSpShRh, FI6-CcSpShRh, and FI7-CcSpShRh. Catchment sizes ranged from 0.5 ha (US4-Cc and US5-CcRh) to 785 ha (CA3-Sc).

Forest harvest methods and subsequent forest operations varied among the sites. For example, forest harvesting was done manually using a chainsaw (US1-Cc, US2-Cc, US3-Cc, US4-Cc, and US5-CcRh) or by mechanized harvesting operations (all the rest). Cable logging was used in US1-Cc, US2-Cc, and US3-Cc. Heavy forestry machines, including harvesters and forwarders, were used in the Fennoscandian and Canadian sites. The harvest occurred on frozen ground in US4-Cc, US5-CcRh, SE4-CcSp, SE5-CcSp, FI2-CcSpSh, FI3-CcSp, FI4-CcSp, FI5-CcSpShRh, FI6-CcSpShRh, and FI7-CcSpShRh. A detailed description of the forest operations undertaken in each catchment are given in the Supporting Information (Table S2).

### 2.2 Field Observations of Site Characteristics

We identified important site-specific characteristics that are indicative of the relative sensitivity of an area to forest harvest, as evidenced by increased THg and MeHg concentrations in stream waters. Harvesting occurred between 1997 and 2015, i.e., 4–22 years before the field visits of this study. We have thereby restricted this study to include site characteristics that we hypothesize are important in determining the sensitivity to forest harvest and are supposed to be relatively stable over decadal time scales.

All catchments were visited between May 2019 and October 2020 to measure site-specific characteristics and obtain first-hand knowledge of forestry management practices and effects. Information about types of trees present before the harvest, i.e., coniferous, deciduous, or mixed forest, was conveyed by local project leaders (Supporting Information, Table S1). In each catchment, the degree of soil disturbance caused by wheel ruts or mechanical site preparation was categorized into the following five classes: (1) minimal, with no visible signs of forest machinery use; (2) low, where there was evidence of forest machinery use, but the mineral or organic peat soils were not exposed and no wheel ruts held water during wet conditions; (3) medium, with some exposed mineral or peat soils and wheel ruts; (4) high, with exposed mineral or peat soils and wheel ruts that were filled with water during wet conditions; and (5) very high, with heavily exposed mineral or peat soils and deep wheel ruts that were filled with water during medium to wet conditions. The integrated soil disturbance level included the whole harvested area, with a special focus on the most disturbed areas and the lower part of the catchment. Disturbance class refers to the conditions immediately after harvest and were evaluated from conditions during the field observations, photographs, and discussion with local project leaders. Consistent observations were possible because the same person (K. Eklöf) collected the data from all catchments, reducing variability and enhancing comparability of the observations. In addition, one or more of the project leaders at each study site assisted during the field visits.

Riparian characteristics were visually assessed but not quantified during field visits and from maps. At some sites, a riparian buffer (i.e., a strip with forest) was left along the stream (US1-Cc, CA2-Sc, CA3-Sc, and SE5CcSp) or at least part of the stream length (CA1-Cc and FI1-CcSp). The width of these buffer zones varied from ∼5–10 m (US1-Cc and SE5-CcSp) to more than 30 m (CA1-Cc, CA2-Sc, and CA3-Sc) on each side of a stream. Riparian wetlands were present along parts of the stream length in two catchments (CA1-Cc and NO1-Cc), where no machine traffic was permitted or possible and therefore served as a machine-free protection zone.

### 2.3 Soil Sample Collection and Field Measures

Soil samples were collected for chemical analyses from each catchment in three transects along the topographical fall line of the hillslope from the stream. In US4 and US5, there was, however, no stream, as shallow groundwater samples were collected in the original study. All soil samples were collected from the harvested area. Along each transect, samples were collected in three different zones: one in the perennial groundwater discharge areas, one in the intermediate area on the border between groundwater discharge and recharge areas, and one in the perennial groundwater recharge areas. At each location, samples were collected using a stainless steel soil coring tube (⌀ = 23 mm) at two depths: the upper 5 cm of the organic soil horizon and the upper 5 cm of the mineral soil horizon. Samples from the three transects were pooled per zone, i.e., three samples of the organic layer from the discharge area were pooled into one sample and so on. In total, six pooled samples from each catchment were collected, except where the organic layer was too thin or peat was >100 cm thick (see the Supporting Information, Table S3). Single-use nitrile gloves were used during soil sampling. The inner parts of the soil core were collected into new polypropylene Falcon tubes and kept cool and dark during transfer to the laboratory. In the USA and Canada, samples were shipped to the Forestry Sciences Laboratory at the US Department of Agriculture Forest Service Northern Research Station in Grand Rapids (for C, N, and S analysis) and Department of Soil Science at the North Dakota State University (NDSU; for Hg analysis). In Fennoscandia, samples were shipped to the soil laboratory at the Swedish University of Agricultural Sciences for C, N, S, and Hg analysis. Soil samples were stored at −18 °C or below until freeze-drying.

The depth of the organic layer, including both O-horizons in podzolic soils and H-horizons in peat soils, was recorded for each core. Mean depths of the organic layer were calculated from each catchment for the groundwater discharge area (*n* = 3), intermediate area (*n* = 3), and recharge area (*n* = 3), respectively.

Stone and boulder content in the soils was measured in each catchment using the Virós surface penetration method.^48–50^ The measurements (*n* = 12) were performed in groundwater recharge areas of harvested areas. Depths of stones or boulders were inferred as obstacles that were encountered when driving a 0.01 m-thick metal rod through soil using uniform striking force and a 2 kg sledge hammer (see detailed description in the Supporting Information, Text S1). The maximum penetration depth was 0.30 m from the mineral soil surface, and depths >0.30 m were all set to 0.31 m. In sites FI2-CcSpSh, FI3-CcSp, FI4-CcSp, FI5-CcSpShRh, FI6-CcSpShRh, and FI7-CcSpShRh, the rod did not reach the mineral layer, and no obstacles were encountered in the peat.

### 2.4 Laboratory Analyses

Chemical analyses of the soil samples included THg concentrations and mass percent of C, N, and S. Samples were freeze-dried and homogenized before analysis. At the USDA Forestry Science Laboratory, soil C and N contents were measured using a Leco CHN 628 total combustion analyzer (standard method at 950 °C/850 °C), and the S content was measured with a Leco Truspec Sulfur module (standard method at 1350 °C). Analyses of C and N at SLU were performed using a Leco Trumac CN instrument, and analyses of S were performed at SLU using ICP-OES after extraction with HNO_3_. Both the lab at NDSU and SLU measured THg concentrations in the soils with a Direct Hg Analyzer (Milestone DMA-80), following the U.S. EPA method 7473.^51^ The method includes a thermal decomposition step, followed by amalgamation and atomic absorption spectrophotometric detection. Quality assurance and quality control data of THg analyses were determined using certified reference materials (Supporting Information, Table S4).

### 2.5 Other Geographical Information Sources

Catchments as well as harvested areas in catchments were delineated manually from topographic maps using GIS mapping (ArcMap 10.7.1). The harvested areas within each catchment were delineated from aerial photographs or from GIS maps provided. The harvested area proportion was calculated in relation to the catchment area (% clear-cut). The hillslope gradient of the harvested area was calculated by dividing the elevation differences by the distance between the highest and lowest point of the harvested area based on digital elevation models. Mean elevation above the stream was used as a proxy for waterlogging, with lower elevations being expected to be more waterlogged than higher elevations. We used DEMs and the tool “Elevation above stream” from the software WhiteboxTool version 1.4.0 (Whitebox Geospatial Inc.) in ArcGIS to calculate elevation above stream for the harvested part of the catchment following Lindsay (2016).^52^

### 2.6 Data Treatment and Statistics

In contrast to the original studies, we collected data only for the harvested catchments, not the corresponding reference catchments. However, the forest harvesting effects on MeHg and THg concentrations in stream or ground/soil water were assessed in the original studies through a comparison of harvested to reference areas. In the original publications, forest harvesting effects were evaluated using different experimental designs. Some of the original studies used a Before–After Control-Impact (BACI) design (US4-Cc, US5-CcRh, CA1-Cc, NO1-Cc, SE1-Lt, SE4-CcSp, SE5-CcSp, SE6-Cc, FI1-CcSp, FI2-CcSpSh, FI3-CcSp, FI4-CcSp, FI5-CcSpShRh, FI6-CcSpShRh, and FI7-CcSpShRh) to isolate the effect of the various treatments from natural variability.^53^ Other studies (US1-Cc, US2-Cc, US3-Cc, CA2-Sc, CA3-Sc, SE2-CcSp, SE3-CcSh, and SE7-Cc) compared concentrations in harvested areas with the concentrations in unharvested areas using a Control-Impact (CI) design. Our findings sometimes differ from original studies related to data handling and approaches to data comparisons. See the Supporting Information (Text S2) for further details regarding treatment of data from original studies.

Orthogonal projections to latent structures (OPLS) analysis, a modification of partial least-squares (PLS) regressions,^54^ was conducted in the software SIMCA 17 (Sartorius Stedim Data Analytics AB, Umeå, Sweden) to analyze which catchment characteristics best explain the variation in effects of forest harvesting on THg and MeHg concentrations in stream waters among the catchments. In these analyses, we did not consider whether statistical effects of forest harvests were reported in the original studies. Instead, we normalized the MeHg concentrations to THg concentrations (ΔMeHg/THg) and THg concentrations to total organic carbon (TOC) concentrations (ΔTHg/TOC), in stream waters and compared these ratios between pre-harvest (before harvest or unharvested reference catchments) and post-harvest conditions. Sites with data below the detection limit for the dependent variables were excluded from the ΔMeHg/THg (*n* = 7) and ΔTHg/TOC (*n* = 5) OPLS analyses (see the Supporting Information, Table S3). To make sure that potential changes in THg/TOC after harvest are not an artifact of changes in TOC, we compared results to an OPLS model on the effect on THg concentrations in stream waters. The strength of the OPLS regression is explained by the goodness of fit *R* ^2^ (the explained variation) and the goodness of prediction *Q* ^2^ (the predicted variation). An OPLS model in which *Q* ^2^ is close to *R* ^2^ implies a model that works well for predicting the response data. Cross-validation was used by the software (Simca 17) to determine the numbers of components to be included and to calculate the predictive power (Q^2^) of the model. The importance of each individual variable in the model is quantified by the variable influence on projection (VIP), where VIP > 1 are commonly identified as most influential for the model.^55^ While the VIP values identify the importance of the variable, the coefficients identify the direction of the relationship between the explanatory and dependent variables.

## 3 Results and Discussions

### 3.1 Forest Harvesting Effects on MeHg Formation and Mobilization

The OPLS model of the spatial variation in ΔMeHg/THg concentrations in stream water had one component that explained 43% of the variation. The low explanatory and predicting power (*R* ^2^ = 0.43, *Q* ^2^ = −0.15) was not surprising, as we have data from rather few sites (*n* = 16) with rather many explanatory variables (*n* = 13). Also, some site characteristics may not be linearly related to ΔMeHg/THg. Despite the lower predictive and explanatory power of the OPLS model, it does indicate which variables are influential for the variation in ΔMeHg/THg concentrations in stream waters. Additionally, these influential variables were further supported by individual plots of each explanatory variable versus the ΔMeHg/THg (Supporting Information, Figure S2), as well as by comparing the significant forestry responses reported in the original studies (Table 1).

**Table 1:** Pre-harvest Concentrations, from before Harvest (BACI Design) or from Unharvested Reference Catchments (CI Design), and Significant Forest Harvesting Effects (% Increases or Decreases) in THg or DHg, and MeHg Concentrations in Stream Waters in the 23 Catchments^a^

catchment	preharvest [THg] or [DHg] (ng/L)	preharvest [MeHg] (ng/L)	harvesting effect [THg] or [DHg] (%)	harvesting effect [MeHg] (%)	hillslope gradient (°)	harvested proportion (%)	disturbance classification (1–5)
US1-Cc	0.3	<0.05	21	0	14.4	93	1
US2-Cc	0.3	<0.05	41	0	20.7	93	1
US3-Cc	0.3	<0.05	21	0	16.4	86	1
US4-Cc	28.1	<0.05	–20	0	11.1	100	1
US5-CcRh	21.2	<0.05	–26	0	11.1	100	1
CA1-Cc	6.5	0.15	0	0	1.4	90	4
CA2-Sc	N/A	0.09	N/A	54	4.8	97	2
CA3-Sc	N/A	0.10	N/A	0	3.4	32	1
NO1-Cc	5.3	0.18	0	0	3.1	38	3
SE1-Lt	3.6	0.05	21	340	8.7	4	5
SE2-CcSp	4.5	0.7	56	86	7.5	65	3
SE3-CcSh	4.5	0.7	0	0	8.0	29	4
SE4-CcSp	3.8	0.24	31	49	2.5	64	3
SE5-CcSp	4.3	0.34	27	0	4.2	35	2
SE6-Cc	7.4	0.22	45	73	3.7	28	5
SE7-Cc	7.1	0.53	0	0	0.9	70	4.5
FI1-CcSp	8.1	0.15	48	133	1.1	92	5
FI2-CcSpSh	6.4	1.17	37	0	2.3	31	5
FI3-CcSp	5.7	0.39	0	0	1.3	24	3
FI4-CcSp	7.7	0.66	0	0	0.2	36	3
FI5-CcSpShRh	6.4	0.19	0	0	0.6	100	4
FI6-CcSpShRh	3.6	0.28	0	0	0.6	42	5
FI7-CcSpShRh	7.9	3.19	0	0	1.6	75	5

a All the aqueous THg and MeHg concentrations and the significant forest harvesting effects are from the original studies (Supporting Information, Table S1). The hillslope gradient was calculated as the slope between the highest and lowest point in the harvested area. The proportion of the catchment that was harvested is a percentage of the full catchment. The soil disturbance classification from after the forest harvest ranges from 1 to 5, where 5 is the highest degree of disturbance.

The variables that best explained the variation in ΔMeHg/THg and that were related to higher values were thick organic soil layers, high pre-harvest aqueous THg or DHg concentrations, and high soil N contents (VIP > 1; Figure 2 Supporting Information, Table S6). These variables described the boreal, peat-rich catchments with a low soil bearing capacity. The importance of high pre-harvest aqueous THg or DHg concentrations in the OPLS model indicates that a large source of inorganic Hg is related to a higher risk of elevated MeHg formation after harvest. Catchments with a higher disturbance classification also had higher ΔMeHg/THg values. Catchments with high slopes and high elevations above the stream had lower ΔMeHg/THg values. The catchments with higher elevation above stream are better drained, where harvest may not have resulted in as much disturbance and new MeHg formation as in the lower gradient catchments.

**Figure 2:**
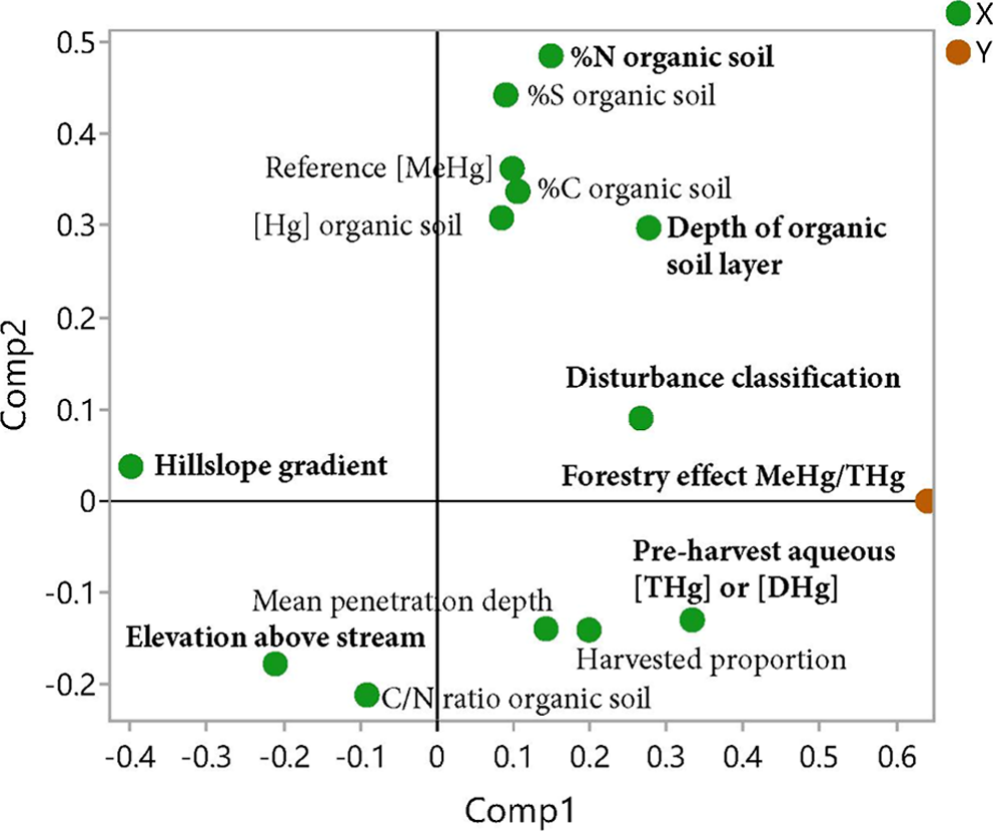
First two components in the OPLS models explaining the variation across the catchments in the change of MeHg/THg in stream waters from before (or reference) conditions to after forest harvest. For illustrative purposes, we forced the model to include two components, even though the best model included one component. The variables with VIP > 1 are the most influential in the model and are bolded.

#### 3.1.1 Soil Disturbance Classification

The soil disturbance classification after harvest had a VIP above 1.11 in the OPLS analyses (Supporting Information, Table S6). Wheel ruts and compaction from forestry equipment may increase the risk of elevated MeHg concentrations in stream waters by creating zones of MeHg formation and by increasing the risk of overland flow.^15,16^ Creation of water-saturated depressions during harvesting supports anoxic conditions with potentially high access to oxidized sulfur and iron compounds (electron acceptors) and labile organic carbon, as energy sources (electron donors) for Hg methylators, such as sulfate-reducing bacteria, iron-reducing bacteria, and methanogens.^14,16,31^ Disturbance classifications were lowest in the catchments where logging had been done manually or with small machinery (US1-Cc, US2-Cc, US3-Cc, US4-Cc, and US5-CcRh) and where selective cutting occurred instead of clear-cutting (CA3-Sc). Of the six catchments with a significant increase of MeHg concentrations in stream waters after forest harvest, three of the catchments (SE1-Lt, SE6-Cc, and FI1-CcSp) had the highest soil disturbance classification (5) after the forestry operations (Table 1). However, this impact was not consistent, as some catchments where MeHg concentrations in stream waters did not increase after forest harvest was characterized with a high disturbance classification (FI2-CcSpSh, FI6-CcSpShRh, and FI7-CcSpShRh). The catchments FI2-CcSpSh, FI6-CcSpShRh, and FI7-CcSpShRh were all harvested peatlands in central Finland, where the harvest, subsequent site preparation, and/or stump harvest caused water-filled depressions from the forestry equipment. Elevated water concentrations of MeHg in ruts and stump hollows were observed in studies in Finland^31^ and Sweden.^16^ These studies^16,31^ suggest that the degree of disturbance can increase the risk for elevated MeHg concentrations in stream waters after forest harvesting and subsequent site preparation and/or stump harvest, but heavy disturbance does not necessarily result in elevated concentrations of MeHg in runoff water.

#### 3.1.2 Depth of the Organic Soil Layer and Mean Elevation above Stream

The degree of soil disturbance can be influenced by the type of forestry equipment used and also by soil bearing capacity. With OPLS, we found a higher organic horizon depth to be positively related and a higher elevation above stream to be negatively related to ΔMeHg/THg in stream waters. In peat-rich and wet areas, the risk of soil disturbance may be higher compared to in drier and mineral soils. Although MeHg/THg increased with increasing depth of the organic layer, it should be noted that the harvest in the Finnish catchments FI2-CcSpSh, FI3-CcSp, FI4-CcSp, FI5-CcSpShRh, FI6-CcSpShRh, and FI7-CcSpShRh, with >100 cm peat, did not increase the MeHg concentrations in ditch water in the original study (Table 1).^31^ A high content of stones and boulders could potentially also increase the soil bearing capacity.^23^ However, the mean penetration depth was not related to ΔMeHg/THg in the OPLS analyses.

#### 3.1.3 Hillslope Gradient

Kronberg et al.^14^ found that newly formed discharge areas, in previously well-drained soils, were the most sensitive part of a harvested hillslope in terms of MeHg formation. Also studies of other land-use activities have found new inundation of formerly unsaturated terrestrial soils, e.g., by beaver dams^56^ and experimental flooding,^57,58^ to be areas of high MeHg formation, potentially due to the high access of readily available electron donors and acceptors.

The expansion of the near-stream discharge area after harvest largely depends on the hillslope gradient of the harvested area and the hydrological response following tree removal. The OPLS analyses demonstrate that low hillslope gradient was related to higher ΔMeHg/THg in stream waters, possibly because there is a higher likelihood of a expansion of the discharge areas after forest harvest. Although there was a negative relationship between hillslope gradients and ΔMeHg/THg, some of the flat catchments (<1°) did not increase in MeHg concentrations in stream waters after harvest (SE7-Cc, FI4-CcSp, FI5-CcSpShRh, and FI6-CcSpShRh; Table 1). In a catchment with low hillslope gradient, the existing discharge area before harvest may be already large, resulting in relatively little expansion of the discharge area and additional influence on the stream water. Also, if concentrations of MeHg are high in stream waters prior to forest harvest, which may be the case in lowland areas with extensive riparian zones, forest harvesting effects are less detectable.^38^ Even if forestry activities create MeHg formation hotspots, these areas may not affect the stream water if the MeHg formation hotspots are not hydrologically connected to the stream.^16,38^ The hydrological connectivity, i.e., the water mediated transport of MeHg from the place where it is formed to the stream, is an important feature determining the amount of MeHg that enters the stream.^59,60^ In the flat (0–2°) peat catchments FI2-CcSpSh, FI3-CcSp, FI4-CcSp, FI5-CcSpShRh, FI6-CcSpShRh, and FI7-CcSpShRh, MeHg hotspots were created in water-filled wheel ruts and holes created by forestry equipment, but low mobilization of MeHg in these flat areas seems to have prevented MeHg from reaching the ditch draining the catchment.^31^ In the catchments US1-Cc, US2-Cc, and US3-Cc, the steep (15–20°) well-drained hillslopes may result in high runoff rates that could promote MeHg mobilization to the stream but may be counteracted by small areas of elevated MeHg formation^25^ (Figure 3). The slopes of the six catchments with significant forest harvesting effects ranged between 1.1° and 8.7°, with a mean value of 4.7° (Table 1 and Supporting Information, Figure S1). It should be noted that the lack of MeHg formation in the very steep (>15°) catchments of US1-Cc, US2-Cc, and US3-Cc could also be related to the low impact logging operations as harvesting was done by a combination of ground and cable logging and forestry equipment was confined to a pre-existing road network without impeding on harvested areas. In addition, these catchments had substantial amounts of logging residue that protected the soil from raindrop erosion and from the direct disturbances occurring during the cable logging operations.^25^

**Figure 3:**
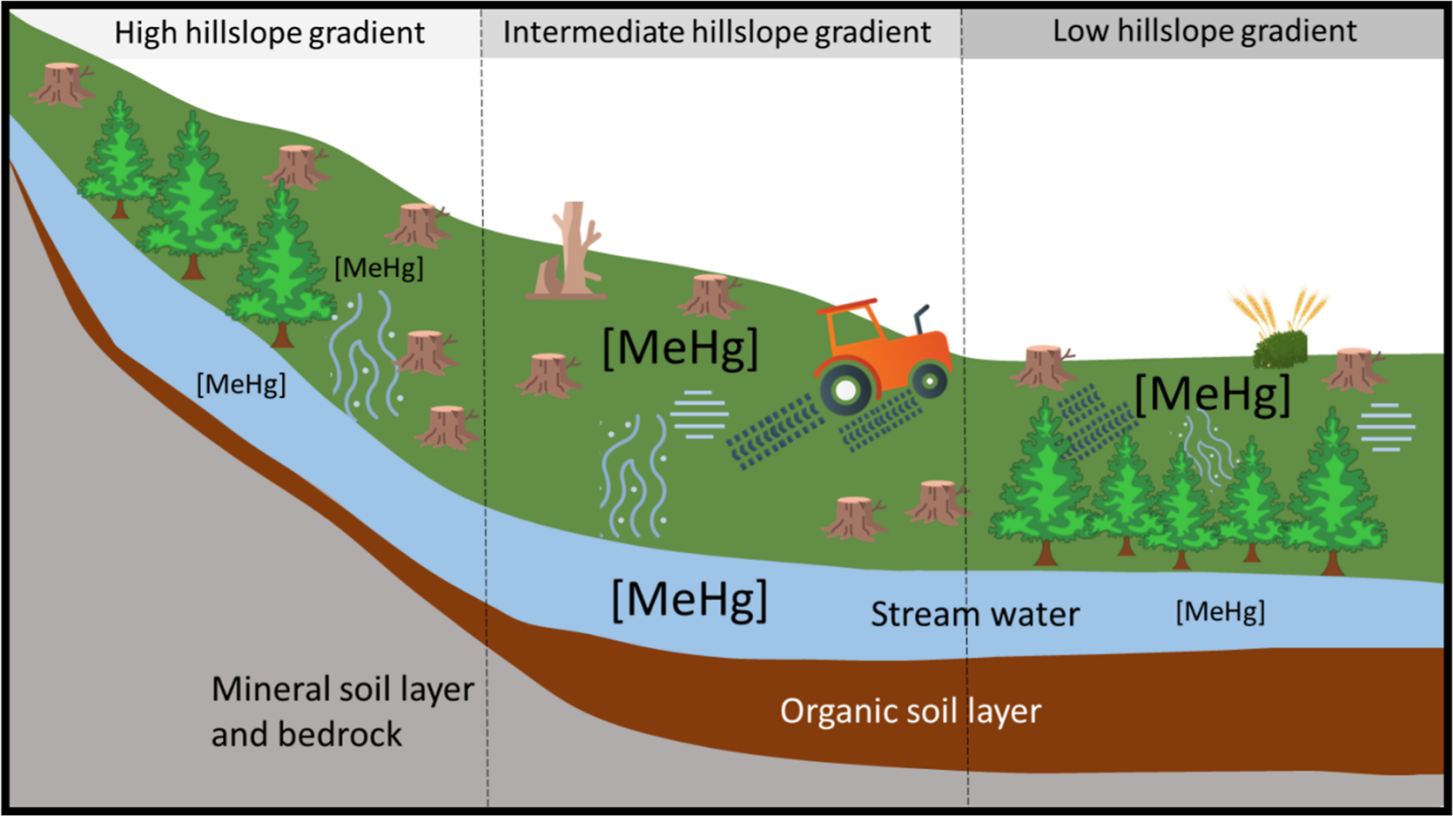
A conceptual figure of the methylmercury (MeHg) formation and mobilization. In catchments with high hillslope gradients (left), forest harvesting leads to low MeHg formation in soil. Consequently, there is little potential MeHg runoff to streams despite high potential water runoff from steep hillslopes. In low hillslope gradients with little elevation above streams (right), the formation rate of MeHg increases with forest harvest, but due to relatively limited change in runoff generation, there is also limited change in MeHg mobilization to streams. A riparian buffer could further lower the water-mediated transport of MeHg to the stream. In intermediate hillslope gradients (middle), the MeHg formation rate may be high due to expansion of near-stream saturated areas after forest harvest compared to higher hillslope gradients. In areas of lower elevation above stream (middle and right), the soil is less well-drained, and if there is peat, the risk of soil disturbance during forest machinery driving is increased. As revealed by OPLS analysis, a high soil nitrogen content and high pre-harvest concentrations of THg in stream waters could increase the risk of high MeHg stream water concentrations after harvest (not depicted in the figure).

#### 3.1.4 Riparian Buffers

Mobilization of MeHg may be influenced by overall hillslope gradients (slope degree) but also by the presence or absence of riparian buffers, i.e., standing trees left after forest harvest (US1-Cc, CA2-Sc, CA3-Sc, SE5-CcSp, and FI1-CcSp; Supporting Information, Table S1) or riparian wetlands with or without trees (CA1-Cc and NO1-Cc; Supporting Information, Table S1). With little riparian disturbance, a riparian buffer may lower the degree of disturbance in the near-stream zone and reduce the risk of MeHg formation hotspots close to the stream. No forestry traffic in the riparian area may prevent overland flow on compacted and/or eroded soils.^61,62^ As riparian buffers were only recorded as absent or present, they were not included in the OPLS analyses. Based on catchment characteristics, CA1-Cc would be expected to have an increase in MeHg stream water concentrations after forest harvest because of intermediate (1.4°) hillslope gradient and high disturbance class (4). However, most of the stream length was surrounded by an approximately 50 m wide riparian wetland. The riparian area likely was the main source of MeHg to the stream, and the uphill contribution was probably insignificant in comparison.^9^ The same is probably true in catchment NO1-Cc, although only part of the stream length was adjacent to the wetlands. In contrast, any harvesting-related disturbance in riparian wetlands may change flow paths and soil conditions, and more MeHg may both be formed after this disturbance and mobilized to the stream.^35^

#### 3.1.5 Soil Nutrient Status

The N content (%) of the soil was positively related to ΔMeHg/THg in the OPLS analyses. The catchments with highest N content (>1.8%; CA1-Cc, FI2-CcSpSh, FI3-CcSp, FI6-CcSpShRh, and FI7-CcSpShRh) all had a C/N of 25 or lower (Supporting Information, Table S5). The C/N may serve as a potential indicator for organic matter quality that can drive MeHg formation.^27,28^ Studies of wetland soils have found the maximum MeHg yield at intermediate soil C/N (∼20).^28^ The C/N may also serve as a potential indicator for vegetation growth that in turn could influence transpiration. If the C/N is high (>25), indicating lower relative N availability, vegetation growth rates may be low.^63,64^ The catchments with C/N > 29 were all from northern Fennoscandia (NO1-Cc, SE4-CcSp, SE5-CcSp, SE7-Cc, FI4-CcSp, and FI5-CcSpShRh). Removal of trees in low productive areas with low timber volume (e.g., NO1-Cc) may have resulted in a smaller change in transpiration after clear-cut compared to the catchments with a high timber volume (e.g., US1-Cc, US2-Cc, and US3-Cc).

#### 3.1.6 Harvest Intensity

The harvested proportion (% clear-cut) of the catchments included in the OPLS regressions varied between 24% (FI3-CcSp) and 100% (US4-Cc, US5-CcRh, and FI5-CcSpShRh; Table 1), but this measure did not influence ΔMeHg/THg in stream waters after harvest. Furthermore, MeHg stream water concentrations at site SE1 with only 4% harvested proportion in the catchment (not included in the OPLS, see the Supporting Information, Text S2) increased by 340% after harvest activities. Although the proportion of harvested area was not correlated to the effect of forest harvesting on MeHg concentrations in stream water, the location of the harvested area within the catchment may be of high importance. If the harvested area is located close to the stream and the sampling site, the influence on stream water response may be larger.

Leaving a proportion of the standing trees contributes to shading. Increased solar insolation in open areas, and higher soil and water temperatures, could potentially increase the formation of MeHg in harvested areas.^30^ Catchments CA2-Sc and CA3-Sc were the only sites where selective cutting was used instead of clear-cutting, with 30–50% of the basal area harvested. When not all trees are removed, the hydrological response may be lower, and there is less risk of erosion and increased mobilization of particle-bound Hg. Selective cutting and lower basal area removal may be reasons why MeHg concentrations in stream waters did not increase after harvest at CA3-Sc (Table 1).

### 3.2 Forest Harvesting Effects on THg Mobilization

The OPLS model of the spatial variation in the ratio between THg (or DHg) and TOC (ΔTHg/TOC) in stream water had two significant components that explained 69% of the variation. As the *R* ^2^ and *Q* ^2^ values were rather close to each other, the model also works well to predict the variation in the data (*R* ^2^ = 0.69, *Q* ^2^ = 0.42). Contrary to the ΔMeHg/THg model, the variation in ΔTHg/TOC was positively related to the elevation above stream and the hillslope gradient and negatively related to the C, N, and S content in the organic layer, the pre-harvest concentrations of THg (or DHg) in stream waters, and the soil disturbance class (Figure 4 and Supporting Information, Table S7). Another study found that increased hillslope gradients and land-use activities can increase the lateral erosion fluxes in terrestrial environments.^65^

**Figure 4:**
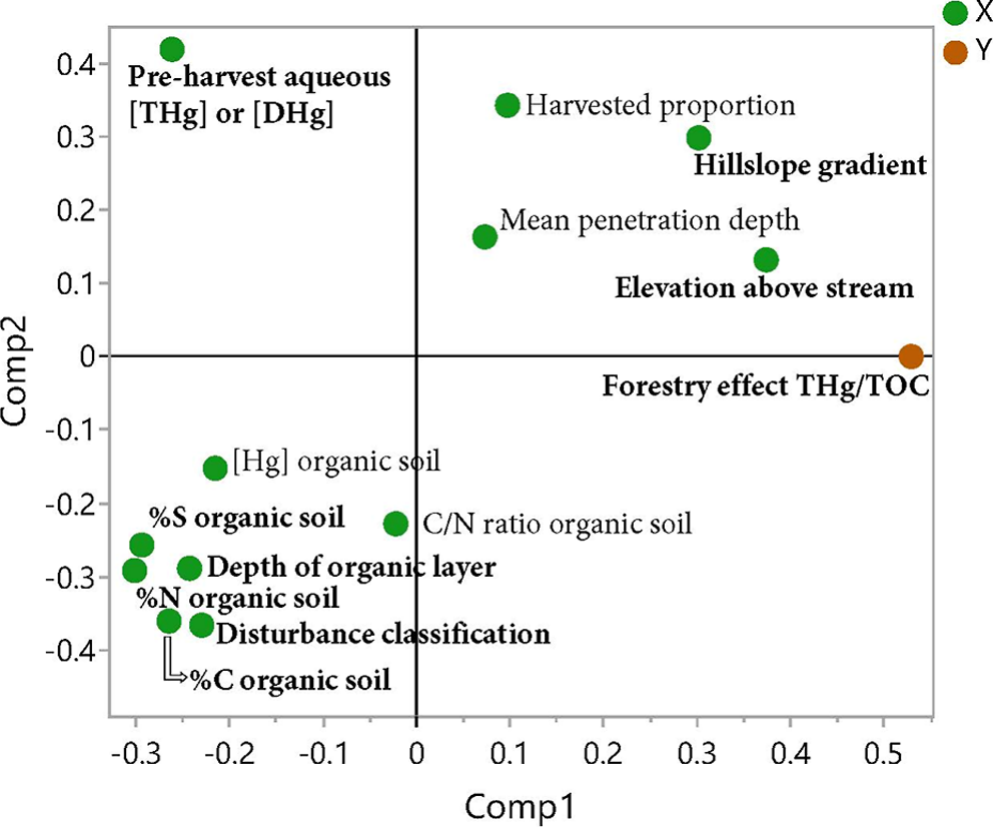
First two components in the OPLS models explaining the variation across the catchments in the change of THg/TOC in stream waters from before (or reference) conditions to after forest harvest. The best model included two components. The variables with VIP > 1 are most influential in the model and are bolded.

The OPLS model with significant increases in THg stream water concentrations as the dependent variable explained 37% of the variation (*R* ^2^ = 0.37, *Q* ^2^ = 0.01; Figure 4 and Supporting Information, Table S7). The less effective model and lower predictability for THg concentrations in stream waters may be explained by less absolute variability in the dependent variable, as many sites showed no increase. Yet, this model included simular explanatory variables as the model with ΔTHg/TOC as the dependent variable. Increases of THg concentrations in stream waters were positively related to the hillslope gradient and elevation above stream and negatively related to the C, N, and S content in the organic horizon, the depth of the organic horizon, and the pre-harvest concentrations of THg (or DHg).

In the catchments where forest harvesting increased THg concentrations in stream waters (*n* = 10 of 21), the increase varied between 21 and 56% (Table 1). The magnitude of increase varied less between catchments for THg concentrations as compared to MeHg concentrations. Among the catchments with no significant increase in THg concentrations, most were boreal peatlands located in central Finland (*n* = 5). These areas were flat (0–2°) with peat >100 cm and minimal erosion after harvest. The stream water THg concentrations in these catchments were not affected, although the soil disturbance classes were high (3–5; Table 1). The soil disturbance class also was negatively related to ΔTHg/TOC in the OPLS analyses, which is contrary to observations for MeHg stream water concentrations. For example, in catchments US1-Cc, US2-Cc, and US3-Cc, DHg concentrations increased by 21–41% after harvest, although the disturbance class was the lowest. The particulate Hg concentrations did not increase in the harvested catchments.^25^ The increase in DHg concentrations may have resulted from increased soil organic matter degradation and subsequent mobilization of Hg bound to DOC or due to changes in soil chemistry and hydrological flow paths as a result of the forest harvesting.^25^ Although MeHg concentrations in stream waters did not increase in the high productivity sites US1-Cc, US2-Cc, and US3-Cc, the hydrological response detected after harvest^25^ may have caused shallower groundwater flow paths as compared to before harvest. As Hg concentrations in soils are commonly higher near the soil surface, shallower groundwater flow paths can mobilize more soil-bound Hg.^18,66^

### 3.3 Are We Comparing Apples and Oranges?

We identify several site characteristics that may increase or decrease the risk of elevated MeHg and THg concentrations in surface waters after forest harvest. This comprehensive quantitative analysis was further enhanced by qualitative information obtained from visiting each catchment, observing differences in site characteristics and forestry operations, and assessing how these factors influence the risk of elevated MeHg formation and mobilization. The variation in forest harvesting effects on responses of THg and MeHg concentrations in receiving waters has been discussed in many former studies.^e.g.^ ^9^ ,^15^ ,^25^ ,^38^ After visiting all the catchments, however, this variation is not that surprising as differences in site characteristics were high, and the variation in forest harvest methods (e.g., cable logging versus ground-based methods) and subsequent forestry activities (e.g., site preparation, stump harvest, logging residue removal, or none) was even higher. Differences in forest harvesting practices and mitigation measures all created different conditions for Hg cycling. More specifically, the risk of MeHg and THg mobilization generally increased with the use of heavy forestry equipment, as in the Fennoscandia sites, as it increases the risk of soil disturbance and compaction, especially in peat soils.

In addition, the risk of MeHg formation increased with

- Forest harvesting of drained peatlands, as in Fennoscandia (e.g., FI2-CcSpSh, FI3-CcSp, FI4-CcSp, FI5-CcSpShRh, FI6-CcSpShRh, and FI7-CcSpShRh) with low bearing capacity where forestry equipment may cause water filled pits and ruts.
- Site preparation after logging that increases standing water, as in many sites in Fennoscandia (e.g., SE4-CcSp, FI2-CcSpSh, FI3-CcSp, FI4-CcSp, FI5-CcSpShRh, FI6-CcSpShRh, and FI7-CcSpShRh).
- The harvest of logging residues (as at US5-CcRh) and/or stumps (as at SE3-CcSh, FI5-CcSpShRh, FI6-CcSpShRh, and FI7-CcSpShRh) that likely create further soil disturbance.

The observed among-catchment variability in forest harvesting methods created conditions for elevated Hg-methylation and mobilization. Given the results herein, detailed descriptions of the forest management practices are critical inclusions when communicating study results to allow for informed comparisons among studies. Descriptions of land-use activities are also valuable because it may not be possible to apply knowledge across international studies due to local or national policy, practice, and other circumstances that drive management schemes and goals. Small differences in land-management practices may have large effects on the environmental response. Future studies could broaden our insight on how to mitigate negative effects across diverse forest management practices and settings and to provide quantitative results from enough studies that would allow the development of numerical criteria and thresholds.

Although there was a large variation in forest harvesting methods among the catchments, we identified factors that inform strategies to mitigate effects of forest harvesting on MeHg concentrations in stream waters. Namely, preventing the formation and mobilization of MeHg is likely of greater importance than preventing the mobilization of THg to surface waters as MeHg is readily available for bioaccumulation in aquatic food webs. Mitigation may be especially important in areas with well-developed organic soil layers, high soil wetness, and intermediate hillslope gradients to reduce the risk of elevated MeHg concentrations in surface waters. Practices such as use of logging residues or logging mats, machinery-free buffer zones, and harvesting on frozen soils to protect soils from forestry machinery may be effective in reducing formation and transport of MeHg to streams. Given the complexity of MeHg stream water responses to forestry operations, it is clear that more studies would help to test if selective cutting instead of clear-cutting and other forestry practices reduces the risk of elevated MeHg in surface waters.

## Supplementary Material



## Notes

Location, study design, and forestry operations in original studies; additional description of method determining the stones and boulder content; additional information about the data treatment, quality assurance, and quality control (QA/QC) of Hg analyses; changes in MeHg/THg and THg/TOC concentrations from before to after harvest; geophysical information and soil chemistry data from each site; explanatory variables included in the OPLS model with forest harvesting effects on ΔMeHg/THg, ΔTHg/TOC, and ΔTHg concentrations as a dependent variable; plots of explanatory variables versus increase of MeHg concentrations from original studies, ΔMeHg/THg and ΔTHg/TOC; a conceptual figure of MeHg formation and mobilization; and a loading plot with the first two components in the OPLS models explaining the variation in forest harvesting effects of THg concentrations in stream waters (PDF)

## References

[1] Pacyna E. G., Pacyna J. M., Steenhuisen F., Wilson S. (2006). Global anthropogenic
mercury emission inventory for 2000. Atmos.
Environ..

[2] Hsu-Kim H., Eckley C. S., Achá D., Feng X., Gilmour C. C., Jonsson S., Mitchell C. P. J. (2018). Challenges
and opportunities for
managing aquatic mercury pollution in altered landscapes. Ambio.

[3] Compeau G. C., Bartha R. (1985). Sulfate-reducing bacteria:
Principal methylators of
mercury in anoxic estuarine sediment. Appl.
Environ. Microbiol..

[4] Gilmour C. C., Henry E. A., Mitchell R. (1992). Sulfate stimulation
of mercury methylation
in freshwater sediments. Environ. Sci. Technol..

[5] Fleming E. J., Mack E. E., Green P. G., Nelson D. C. (2006). Mercury
methylation
from unexpected sources: molybdate-inhibited freshwater sediments
and an iron-reducing bacterium. Appl. Environ.
Microbiol..

[6] Bravo A. G., Bouchet S., Guédron S., Amouroux D., Dominik J., Zopfi J. (2015). High methylmercury
production under ferruginous conditions in sediments
impacted by sewage treatment plant discharges. Water Res..

[7] Hamelin S., Amyot M., Barkay T., Wang Y., Planas D. (2011). Methanogens:
Principal Methylators of Mercury in Lake Periphyton. Environ. Sci. Technol..

[8] Bae H.-S., Dierberg F. E., Ogram A., Wommack K. E. (2014). Syntrophs Dominate
Sequences Associated with the Mercury Methylation-Related Gene hgcA
in the Water Conservation Areas of the Florida Everglades. Appl. Environ. Microbiol..

[9] Bishop K., Shanley J. B., Riscassi A., de Wit H. A., Eklöf K., Meng B., Mitchell C., Osterwalder S., Schuster P. F., Webster J., Zhu W. (2020). Recent advances in
understanding and measurement of mercury in the environment: Terrestrial
Hg cycling. Sci. Total Environ..

[10] Åkerblom S., Bignert A., Meili M., Sonesten L., Sundbom M. (2014). Half a century
of changing mercury levels in Swedish freshwater fish. Ambio.

[11] Braaten H. F. V., Åkerblom S., Kahilainen K. K., Rask M., Vuorenmaa J., Mannio J., Malinen T., Lydersen E., Poste A. E., Amundsen P.-A., Kashulin N., Kashulina T., Terentyev P., Christensen G., de Wit H. A. (2019). Improved Environmental
Status: 50 Years of Declining
Fish Mercury Levels in Boreal and Subarctic Fennoscandia. Environ. Sci. Technol..

[12] Lepak J. M., Hooten M. B., Eagles-Smith C. A., Tate M. T., Lutz M. A., Ackerman J. T., Willacker J. J., Jackson A. K., Evers D. C., Wiener J. G., Pritz C. F., Davis J. (2016). Assessing potential
health risks to fish and humans using mercury concentrations in inland
fish from across western Canada and the United States. Sci. Total Environ..

[13] Sikström U., Hökkä H. (2016). Interactions
between soil water conditions and forest
stands in boreal forests with implications for ditch network maintenance. Silva Fennica.

[14] Kronberg R.-M., Jiskra M., Wiederhold J. G., Björn E., Skyllberg U. (2016b). Methyl Mercury Formation in Hillslope
Soils of Boreal
Forests: The Role of Forest Harvest and Anaerobic Microbes. Environ. Sci. Technol..

[15] Eklöf K., Lidskog R., Bishop K. (2016). Managing Swedish forestry’s
impact on mercury in fish: Defining the impact and mitigation measures. Ambio.

[16] Eklöf K., Bishop K., Bertilsson S., Björn E., Buck M., Skyllberg U., Osman O. A., Kronberg R.-M., Bravo A. G. (2018). Formation of mercury
methylation hotspots as a consequence
of forestry operations. Sci. Total Environ..

[17] McCarter C. P. R., Eggert S. L., Sebestyen S. D., Kolka R. K., Mitchell C. P. J. (2022). Effects
of Clearcutting and Residual Biomass Harvesting on Hillslope Mercury
Mobilization and Downgradient Mercury Accumulation. J. Geophys. Res.:Biogeosci..

[18] McCarter C. P. R., Sebestyen S. D., Eggert S. L., Kolka R. K., Mitchell C. P. J. (2021). Differential
subsurface mobilization of ambient mercury and isotopically enriched
mercury tracers in a harvested and residue harvested hardwood forest
in northern Minnesota. Biogeochemistry.

[19] Kozlowski T. T. (1999). Soil Compaction
and Growth of Woody Plants. Scand. J. For. Res..

[20] Eklöf K., Schelker J., Sørensen R., Meili M., Laudon H., von Brömssen C., Bishop K. (2014). Impact of Forestry on Total and Methyl-Mercury
in Surface Waters: Distinguishing Effects of Logging and Site Preparation. Environ. Sci. Technol..

[21] Eklöf K., Meili M., Åkerblom S., von Brömssen C., Bishop K. (2013). Impact of stump harvest on run-off concentrations of
total mercury and methylmercury. For. Ecol.
Manage..

[22] Greacen E. L., Sands R. (1980). Compaction of forest
soils. A review. Soil
Res..

[23] Niemi M. T., Vastaranta M., Vauhkonen J., Melkas T., Holopainen M. (2017). Airborne LiDAR-derived
elevation data in terrain trafficability mapping. Scand. J. For. Res..

[24] Cambi M., Certini G., Neri F., Marchi E. (2015). The impact of heavy
traffic on forest soils: A review. For. Ecol.
Manage..

[25] Eckley C. S., Eagles-Smith C., Tate M. T., Kowalski B., Danehy R., Johnson S. L., Krabbenhoft D. P. (2018). Stream Mercury Export in Response
to Contemporary Timber Harvesting Methods (Pacific Coastal Mountains,
Oregon, USA). Environ. Sci. Technol..

[26] Bravo A. G., Kothawala D. N., Attermeyer K., Tessier E., Bodmer P., Ledesma J. L. J. (2018). The interplay between total mercury, methylmercury
and dissolved organic matter in fluvial systems: A latitudinal study
across Europe. Water Res..

[27] Bravo A., Bouchet S., Tolu J., Björn E., Mateos-Rivera A., Bertilsson S. (2017). Molecular composition of organic
matter controls methylmercury formation in boreal lakes. Nat. Commun..

[28] Tjerngren I., Meili M., Björn E., Skyllberg U. (2012). Eight Boreal
Wetlands as Sources and Sinks for Methyl Mercury in Relation to Soil
Acidity, C/N Ratio, and Small-Scale Flooding. Environ. Sci. Technol..

[29] de
Wit H. A., Granhus A., Lindholm M., Kainz M. J., Lin Y., Braaten H. F. V., Blaszczak J. (2014). Forest harvest
effects on mercury in streams and biota in Norwegian boreal catchments. For. Ecol. Manage..

[30] Sørensen R., Meili M., Lambertsson L., von Brömssen C., Bishop K. (2009). The effect of forest harvest operations
on mercury
and methylmercury in two boreal streams: Relatively small changes
in the first two years prior to site preparation. Ambio.

[31] Ukonmaanaho L., Starr M., Kantola M., Laurén A., Piispanen J., Pietilä H., Perämäki P., Merilä P., Fritze H., Tuomivirta T. (2016). Impacts of forest harvesting
on mobilization of Hg and MeHg in drained
peatland forests on black schist or felsic bedrock. Environ. Monit. Assess..

[32] Allan, C. J. ; Heyes, A. ; Mackereth, R. J. Changes to groundwater and surface water Hg transport following clearcut logging: a Canadian case study. In Does forestry contribute to mercury in Swedish fish? Royal Swedish Academy of Agriculture and Forestry (KSLA) report 2009, 148, 50−54, Stockholm,

[33] Eklöf K., Kraus A., Weyhenmeyer G. A., Meili M., Bishop K. (2012). Forestry influence
by stump harvest and site preparation on methylmercury, total mercury
and other stream water chemistry parameters across a boreal landscape. Ecosystems.

[34] Porvari P., Verta M., Munthe J., Haapanen M. (2003). Forestry practices
increased mercury and methyl mercury output from boreal forst catchments. Environ. Sci. Technol..

[35] Munthe J., Hultberg H. (2004). Mercury and methylmercury
in runoff from a forested
catchment - concentrations, fluxes, and their response to manipulations. Water, Air, Soil Pollut.:Focus.

[36] Skyllberg U., Westin M. B., Meili M., Björn E. (2009). Elevated concentrations
of methyl mercury in streams after forest clear-cut: A consequence
of mobilization from soil or new methylation?. Environ. Sci. Technol..

[37] Munthe J., Hellsten S., Zetterberg T. (2007). Mobilization
of mercury and methylmercury
from forest soils after a severe storm-fell event. Ambio.

[38] Kronberg R.-M., Drott A., Jiskra M., Wiederhold J. G., Björn E., Skyllberg U. (2016a). Forest harvest
contribution to Boreal
freshwater methyl mercury load. Global Biogeochem.
Cycles.

[39] Garcia E., Carignan R. (2000). Mercury concentrations
in northern pike (Esox lucius)
from boreal lakes with logged, burned, or undisturbed catchments. Can. J. Fish. Aquat. Sci..

[40] Willacker J. J., Eagles-Smith C. A., Kowalski B. M., Danehy R. J., Jackson A. K., Adams E. M., Evers D. C., Eckley C. S., Tate M. T., Krabbenhoft D. P. (2019). Timber harvest alters mercury bioaccumulation and food
web structure in headwater streams. Environ.
Pollut..

[41] Garcia E., Carignan R., Lean D. R. S. (2007). Seasonal
and inter-annual variations
in methyl mercury concentrations in zooplankton from boreal lakes
impacted by deforestation or natural forest fires. Environ. Monit. Assess..

[42] Desrosiers M., Planas D., Mucci A. (2006). Short-term
responses to watershed
logging on biomass mercury and methylmercury accumulation by periphyton
in boreal lakes. Can. J. Fish. Aquat. Sci..

[43] Wu P., Bishop K., von Brömssen C., Eklöf K., Futter M., Hultberg H., Martin J., Åkerblom S. (2018). Does forest
harvest increase the mercury concentrations in fish? Evidence from
Swedish lakes. Sci. Total Environ..

[44] Garcia E., Carignan R. (1999). Impact of wildfire
and clear-cutting in the boreal
forest on methyl mercury in zooplankton. Can.
J. Fish. Aquat. Sci..

[45] Rask M., Nyberg K., Markkanen S.-L., Ojala A. (1998). Forestry in catchments:
Effects on water quality, plankton, zoobenthos and fish in small lakes. Boreal Environ. Res..

[46] Charbonneau K. L., Kidd K. A., Kreutzweiser D. P., Sibley P. K., Emilson E. J. S., O’Driscoll N. J., Gray M. A. (2022). Are There Longitudinal
Effects of Forest Harvesting on Carbon Quality and Flow and Methylmercury
Bioaccumulation in Primary Consumers of Temperate Stream Networks?. Environ. Toxicol. Chem..

[47] Eklöf, K. ; Löfvenius, P. ; Meili, M. ; Karlsen, R. H. ; Bishop, K. Mercury in Runoff Water as a Consequence of Forestry: Stem-Only Harvest Versus whole-tree Harvest. In preparation.

[48] Viro, P. J. The mechanical composition and fertility of forest soil taking into consideration especially the stoniness of the soil. Communicationes Instituti Forestalis Fenniae 1947, 35(2) 115 p. (In Finnish with English summary).

[49] Stendahl J., Lundin L., Nilsson T. (2009). The stone and boulder
content of
Swedish forest soils. CATENA.

[50] Eriksson C. P., Holmgren P. (1996). Estimating stone and boulder content
in forest soils
- evaluating the potential of surface penetration methods. Catena.

[51] US EPA . Method 7473 (SW-846): Mercury in Solids and Solutions by Thermal Decomposition, Amalgamation, and Atomic Absorption Spectrophotometry; Revision 0; US EPA: Washington, DC, 2007.

[52] Lindsay J. B. (2016). Efficient
hybrid breaching-filling sink removal methods for flow path enforcement
in digital elevation models. Hydrol. Processes.

[53] Smokorowski K. E., Randall R. G. (2017). Cautions on using the Before-After-Control-Impact design
in environmental effects monitoring programs. FACETS.

[54] Trygg J., Wold S. (2002). Orthogonal projections
to latent structures (O-PLS). J. Chemom..

[55] Eriksson, L. ; Johansson, E. ; Kettaneh-Wold, N. ; Wold, S. Introduction to multi- and Megavariate Data Analysis Using Projection Methods (PCA & PLS); Umetrics AB, Umeå, 1999.

[56] Levanoni O., Bishop K., McKie B. G., Hartman G., Eklöf K., Ecke F. (2015). Impact of Beaver Pond Colonization
History on Methylmercury Concentrations
in Surface Water. Environ. Sci. Technol..

[57] Hall B., Louis V. L. S., Rolfhus K. R., Bodaly R. A., Beaty K. G., Paterson M. J., Cherewyk K. A. P. (2005). Impacts
of Reservoir Creation on
the Biogeochemical Cycling of Methyl Mercury and Total Mercury in
Boreal Upland Forests. Ecosystems.

[58] St.Louis V. L., Rudd J. W. M., Kelly C. A., Drew
Bodaly R. A., Paterson M. J., Beaty K. G., Hesslein R. H., Heyes A., Majewski A. R. (2004). The Rise and Fall of Mercury Methylation
in an Experimental
Reservoir. Environ. Sci. Technol..

[59] Freeman M. C., Pringle C. M., Jackson C. R. (2007). Hydrologic
Connectivity and the Contribution
of Stream Headwaters to Ecological Integrity at Regional Scales^1^. J. Am. Water Resour. Assoc..

[60] McCarter C. P. R., Sebestyen S. D., Jeremiason J. D., Nater E. A., Kolka R. K. (2024). Methylmercury
export from a headwater peatland catchment decreased with cleaner
emissions despite opposing effect of climate warming. Water Resour. Res..

[61] Huang H., Mackereth R. W., Mitchell C. P. (2024). Impacts of forest harvesting on mercury
concentrations and methylmercury production in boreal forest soils
and stream sediment. Environ. Pollut..

[62] Lam W. Y., Mackereth R. W., Mitchell C. P. J. (2024). Mercury concentrations
and export
from small central Canadian boreal forest catchments before, during,
and after forest harvest. Sci. Total Environ..

[63] Högberg M. N., Myrold D. D., Giesler R., Högberg P. (2002). Contrasting
patterns of soil N-cycling in model ecosystems of Fennoscandian boreal
forests. Oecologia.

[64] Högberg P., Wellbrock N., Högberg M. N., Mikaelsson H., Stendahl J. (2021). Large differences in plant nitrogen
supply in German
and Swedish forests – Implications for management. For. Ecol. Manage..

[65] Liu M., Zhang Q., Ge S., Mason R. P., Luo Y., He Y., Xie H., Sa R., Chen L., Wang X. (2019). Rapid Increase
in the Lateral Transport of Trace Elements Induced by Soil Erosion
in Major Karst Regions in China. Environ. Sci.
Technol..

[66] Oswald C. J., Heyes A., Branfireun B. A. (2014). Fate and Transport of Ambient Mercury
and Applied Mercury Isotope in Terrestrial Upland Soils: Insights
from the METAALICUS Watershed. Environ. Sci.
Technol..

