# Supplementary material for: The Effects of Forest Harvesting on Total and Methylmercury Concentrations in Surface Waters Depend on Harvest Practices and Physical Site Characteristics
Source: Environ Sci Technol. 2025 Jul 22;59(30):15944–55. doi: 10.1021/acs.est.5c02787 (PMC12329714; doi:10.1021/acs.est.5c02787)
Supplement: Supplementary file 1 [file es5c02787_si_001.pdf]

Supplementary material

Effects of forest harvesting on total- and methylmercury concentrations in surface waters depend on harvest practices and physical site characteristics

Karin Eklöf[1], Heleen de Wit[2, 3], Chris S. Eckley[4], Collin A. Eagles-Smith[5], Susan L. Eggert[6], Robert W. Mackereth[7], Ulf Skyllberg[8], Liisa Ukonmaanaho[9], Matti Verta[10], Craig Allan[11], Erik J.S. Emilson[12], Karen A. Kidd[13], Carl P.J. Mitchell[14], John Munthe[15], Tapani Sallantaus[10], Joel Segersten[1], Andrea G. Bravo[16], Randall K. Kolka[6], Colin P.R. McCarter[17], Petri Porvari[9], Eva Ring[18], Stephen D. Sebestyen[6], Ulf Sikström[18], and Therese Sahlén Zetterberg[19]

[1]Department of Aquatic Sciences and Assessment, Swedish University of Agricultural Sciences, SE-75007 Uppsala, Sweden

[2]Norwegian Institute for Water Research, Økernveien 94, 0579 Oslo, Norway

[3]Center of Biogeochemistry in the Anthropocene, Department of Biosciences, University of Oslo, P.O. Box 1066, 0371 Oslo, Norway

[4]United States Environmental Protection Agency, Region 10, 1200 6th Ave, Seattle WA 98101 USA

[5]United States Geological Survey, Forest and Rangeland Ecosystem Science Center, 3200 SW Jefferson Way, Corvallis OR 97331 USA

[6]United States Department of Agriculture Forest Service, Northern Research Station, Grand Rapids, MN 55744, USA

[7]Centre for Northern Forest Ecosystem Research, Ontario Ministry of Natural Resources, 421 James St S., Thunder Bay ON P7E 2V6, Canada

[8]Department of Forest Ecology and Management, Swedish University of Agricultural Sciences, SE-90183 Umeå, Sweden

[9]*Natural Resources Institute Finland (LUKE), Latokartanonkaari 9, 00790 Helsinki, Finland*
[10]*Finnish Environment Institute (Syke), Latokartanonkaari 11, 00790 Helsinki, Finland*

[11]*Department of Earth, Environmental and Geographical Sciences, University of North Carolina Charlotte, Charlotte, NC 28223, USA*

[12]*Natural Resources Canada, Great Lakes Forestry Centre, 1219 Queen St. E, Sault Ste. Marie, ON P6A2E3, Canada*

[13]*Department of Biology, McMaster University, Hamilton, ON L8S4K1, Canada*

[14]*Department of Physical and Environmental Sciences, University of Toronto Scarborough, Toronto MIC1A4, ON, Canada*

[15]*IVL Swedish Environmental Research Institute, PO Box 530 21, SE-40014 Göteborg, Sweden*

[16]*Departament de Biologia Marina i Oceanografia, Institut de Ciències del Mar, ICM-CSIC, 08003 Barcelona, Catalunya, Spain*

[17]*Department of Biology, Chemistry, and Geography, Nipissing University, North Bay, ON, P1B8L7, Canada*

[18]*Skogforsk, The Forestry Research Institute of Sweden, Uppsala Science Park, SE-75183 Uppsala, Sweden*

[19]*Department of Soil and Environment, Swedish University of Agricultural Sciences, SE-75007 Uppsala, Sweden.*

*Corresponding author, email: Karin.Eklof@slu.se

Summary: 54 pages, 2 texts, 5 figures, 8 tables

Table of Content

Table S1. Information about the location and study design in the original studies

Table S2. Information about forestry operations performed in each of the 23 sites

Text S1. Description of method for determining stones and boulder content

Text S2. Information about the data treatment

Table S3. Quality assurance and quality control (QA/QC)

Table S4. Changes in ΔMeHg/THg and ΔTHg/TOC from before to after harvest, as well as geophysical information from each site

Table S5. Soil chemistry data from each site

Table S6. The explanatory variables included in the OPLS model with forestry effect on MeHg/THg concentrations as dependent variable

Figure S1. The relationship between the increase of MeHg concentrations from original studies versus hillslope gradient, and soil disturbance classification

Figure S2. The relationship between the change of MeHg/THg stream water concentrations from original studies versus all explanatory variables included in the OPLS analyses

Figure S3. A conceptual figure of the methylmercury (MeHg) formation and mobilization

Table S7. The explanatory variables included in the OPLS model with forestry effect on THg/TOC concentrations as dependent variable

Figure S4. The relationship between the change of THg/TOC stream water concentrations from original studies versus all explanatory variables included in the OPLS analyses

Figure S5. The first two components in the OPLS models explaining the variation in forestry effect of THg concentrations in stream waters

Table S8. The explanatory variables included in the OPLS model with forestry effect on THg concentrations as dependent variable

Table S1. Information about the location of and forestry operations performed (Treatment) in each of the 23 catchments. The study design in each catchment, as presented in the original publications, is either a comparison between impacted (harvested) and control (non-harvested reference) catchments (CI), or a Before-After-Control-Impact (BACI) design. The table also presents the type of forest stand (tree vegetation) and size of machine-free protection zone bordering the streams, if present.

**Catchment ID**	**Location and site name in original publication**	**Treatment***	**Study design**	**Tree vegetation**	**Riparian characteristics**	**Reference to original studies**
US1-Cc	Oregon, USA, PH4	Cc	CI	Coniferous	15 m forest buffer	Eckley et al., 2018
US2-Cc	Oregon, USA, GS3	Cc	CI	Coniferous	No riparian buffer	Eckley et al., 2018
US3-Cc	Oregon, USA, UM2	Cc	CI	Coniferous	No riparian buffer	Eckley et al., 2018
US4-Cc	Minnesota, USA, plot 2, CC	Cc	BACI	Deciduous	No riparian buffer	McCarter et al., 2021
US5-CcRh	Minnesota, USA, plot 1 CC+B	Cc+Rh	BACI	Deciduous	No riparian buffer	McCarter et al., 2021
CA1-Cc	Northwest Ontario, Canada, E1	Cc	BACI	Mixed	50-100 m riparian wetland buffer	Allan et al., 2009
CA2-Sc	Ontario, Canada, Bat3	Sc	CI	Deciduous	30 m forest buffer	Charbonneau et al., 2022
CA3-Sc	Ontario, Canada, Ker4	Sc	CI	Deciduous	30 m forest buffer	Charbonneau et al., 2022
NO1-Cc	Norway, Langetjern	Cc	BACI	Coniferous	0-30 m riparian wetland buffer	de Wit et al., 2014
SE1-Lt	Southwestern Sweden, Gårdsjön	Lr	BACI	Coniferous	No riparian buffer	Munthe and Hultberg, 2004
SE2-CcSp	South Sweden, SP	Cc+Sp	CI	Coniferous	No riparian buffer	Eklöf et al., 2013
SE3-CcSh	South Sweden, SH1	Cc+Sh	CI	Coniferous	No riparian buffer	Eklöf et al., 2013
SE4-CcSp	North Sweden, CC	Cc+Sp	BACI	Coniferous	No riparian buffer	Eklöf et al., 2014 Sørensen et al., 2009
SE5-CcSp	North Sweden, North	Cc+Sp	BACI	Coniferous	5-10 m forest buffer	Eklöf et al., 2014 Sørensen et al., 2009
SE6-Cc	Northern Sweden, Strömsjöliden	Cc	BACI	Coniferous	No riparian buffer	Eklöf et al. in prep.

SE7-Cc	North Sweden, below ML, CC4 and CC2 (mean values)	Cc	CI	Coniferous	No riparian buffer	Kronberg et al., 2016a; Kronberg et al., 2016b
FI1-CcSp	Southern Finland	Cc+Sp	BACI	Coniferous	0-20 m forest buffer	Porvari et al. 2003
FI2-CcSpSh	Central Finland, FS_WTHs2	Cc+Sp+Sh	BACI	Coniferous	No riparian buffer	Ukonmaanaho et al., 2016
FI3-CcSp	Central Finland, FS_SOH	Cc+Sp	BACI	Coniferous	No riparian buffer	Ukonmaanaho et al., 2016
FI4-CcSp	Central Finland, BS_SOH	Cc+Sp	BACI	Coniferous	No riparian buffer	Ukonmaanaho et al., 2016
FI5-CcSpShRh	Central Finland, FS_WTHs1	Cc+Sp+Sh+Rh	BACI	Coniferous	No riparian buffer	Ukonmaanaho et al., 2016
FI6- CcSpShRh	Central Finland, BS_WTHs1	Cc+Sp+Sh+Rh	BACI	Coniferous	No riparian buffer	Ukonmaanaho et al., 2016
FI7-CcSpShRh	Central Finland, BS_WTHs2	Cc+Sp+Sh+Rh	BACI	Coniferous	No riparian buffer	Ukonmaanaho et al., 2016

*Clear-cutting (Cc), Selective cutting (Sc), Mechanical site preparation (Sp), Stump harvest (Sh), Logging residue harvest (Rh), and Logging trail (Lt)

Table S2. Information about the forest management performance in each catchment as well as the sample frequency and number of years in the original studies.

Catchment	Forest management performance	Sample frequency original study (number of years before/after harvest)
US1	Forest harvest was conducted using a combination of ground and cable logging during summer conditions. Forest machinery did not drive in the harvested areas, others than on gravel roads, that were upgraded or constructed prior to harvest. Logging residues were left on site after harvest.	Monthly (1 year after harvest)
US2	Forest harvest was conducted using a combination of ground and cable logging during summer conditions. Forest machinery did not drive in the harvested areas, others than on gravel roads, that were upgraded or constructed prior to harvest. Logging residues were left on site after harvest.	Monthly (1 year after harvest)
US3	Forest harvest was conducted using a combination of ground and cable logging during summer conditions. Forest machinery did not drive in the harvested areas, others than on gravel roads, that were upgraded or constructed prior to harvest. Logging residues were left on site after harvest.	Monthly (1 year after harvest)
US4	Hillside forest was clearcut harvested and delimbed with hand chainsaw and logs mechanically skidded over frozen soils and relatively shallow snow cover to roadside landing. Residual biomass was left on the forest floor. Skidder was excluded from riparian peatland area.	Weekly sampling during spring, summer, and fall with higher resolution sampling during precipitation events and snowmelt. (2 years before and 1.75 years after harvest).
US5	Hillside forest was clearcut harvested with hand chainsaw and entire trees mechanically skidded over frozen soils and relatively shallow snow cover to roadside landing. Approximately 85% of residual biomass was removed from the site by machine and by hand after forest harvesting. Skidder was excluded from riparian peatland area.	Weekly sampling during spring, summer, and fall with higher resolution sampling during precipitation events and snowmelt. (2 years before and 1.75 years after harvest).
CA1	Forest harvest was carried out with a feller buncher in June and logs were skidded to the adjacent roadside for limbing and transport. Skidders were excluded from riparian wetland areas (approximately 50m wide).	Biweekly during the growing season and more frequently during snowmelt. (1.5 years before and 2.5 years after harvest)
CA2	Forest harvest was mainly selection-based cutting, with a rate of 30 to 50% basal area removal, and only small areas of clearcutting. A harvester and forwarder were used. Sampling started within 5 years after the harvest.	Three times a year (2 years after harvest)
CA3	Forest harvest was mainly selection-based cutting, with a rate of 30 to 50% basal area removal, and only small areas of clearcutting. A harvester and forwarder were used. Sampling started within 5 years after the harvest.	Three times a year (2 years after harvest)
NO1	Forest harvest was carried out using a harvester and forwarder in January. The harvest was scheduled for winter to be done on frozen soils, but due to mild weather the ground was not frozen. The harvest operations thereby resulted in wheel ruts, especially in the lower and wetter parts of the catchment.	Biweekly to monthly (0.5 year before and 3 years after harvest)

SE1	No harvest was conducted in this catchment, and this catchment is not included in the multivariate statistics in this study. The catchment is, however, discussed in this study. Heavy forestry machinery (tractors and transport vehicles) caused disturbance of the forest soils and a temporary logging road was created through the catchment. Soil and branches used to stabilize the logging road blocked the ditch and caused a <150 m^2 inundation upstream of the logging road. The blockage was removed within a weeks, but the soil conditions remained altered.	Biweekly to monthly (6 years before and 3 years after logging road)
SE2	Forest harvest was carried out using a harvester and forwarder during summer conditions. In autumn the following year the catchment was stump harvested. Stump harvest was performed by forestry machinery that extracts the stumps and then cuts and shakes them to remove excess soil from the root system.	Biweekly (3 years after harvest)
SE3	Forest harvest was carried out using a harvester and forwarder during summer conditions. During unfrozen conditions in January, the catchment was site prepared.	Biweekly (3 years after harvest)
SE4	Forest harvest was carried out using a harvester and forwarder during winter on soils covered with snow. All the harvested areas were site prepared by disc trenching when the soil was free of snow and soil frost. The disc-trencher was fitted with sowing equipment. Scots pine seeds were distributed in the clear-cut area.	Biweekly (1 year before and 5 years after harvest)
SE5	Forest harvest was carried out using a harvester and forwarder during winter on soils covered with snow. Wetlands were not harvested and harvesting within 10 m of the stream was avoided. All the harvested areas were site prepared by disc trenching when the soil was free of snow and soil frost.	Biweekly (1 year before and 5 years after harvest)
SE6	Forest harvest was carried out using a harvester and forwarder during summer conditions. Stump harvest was carried out one year later, also during summer conditions. Stump harvest was performed with an excavator that extracts the stumps and then cuts and shakes them to remove excess soil from the root system.	Biweekly (Almost 2 years before and a little more than 2 years after harvest)
SE7	Forest harvest was carried out using a harvester and forwarder. In one of the catchments (CC2 below marine limit (ML)), site preparation was completed by drag scarification and in the other catchment (CC4 below ML) by mounding.	Sampling was done every 1.5 months from May 2011 to April 2012. The harvest were conducted 2 years prior to the sampling.
FI1	Forest harvest was carried out using a harvester and forwarder during summer conditions (Aug-Sept). Site preparation by mounding was completed in September, two years after the harvest.	Monthly at base flow and more frequently at high flow (3 years before and 3 years after harvest)
FI2	Forest harvesting and logging residue removal were conducted using harvester and forwarder in late winter when the area was snow covered. Stump harvest was carried out in early autumn the same year with an excavator. During stump harvest, the ground was not frozen; therefore, deep ruts were formed locally on the harvested areas. Site preparation (mounding) was conducted in the harvested area during summer conditions 1.5 years after harvest.	During the calibration year, sampling was conducted 4 times a year. In following years, sampling was conducted monthly during the snow free period (1 year before and 4 years after harvest)
FI3	Forest harvesting was conducted using harvester and forwarder in late winter when the area was snow covered. Site preparation (mounding) was conducted in the harvested area during summer conditions 1.5 years after the harvest.	During the calibration year sampling was conducted 4 times a year. In following years, sampling was conducted monthly during the snow free period (1

		year before and 4 years after harvest)
FI4	Forest harvesting was conducted using harvester and forwarder in late winter when the area was snow covered. Site preparation (mounding) was conducted in the harvested area during summer conditions 1.5 years after the harvest.	During calibration year sampling was conducted 4 times a year. In following years, sampling was conducted monthly during snow free period (1 year before and 4 years after harvest)
FI5	Forest harvesting and logging residue removal were conducted using harvester and forwarder in late winter when the area was snow covered. Stump harvest was carried out in early autumn the same year with an excavator. During stump harvest, the ground was not frozen; therefore, deep ruts were formed locally on the harvested areas. Site preparation (mounding) was conducted in the harvested area during summer conditions 1.5 years after the harvest.	During calibration year sampling was conducted 4 times a year. In following years, sampling was conducted monthly during snow free period (1 year before and 4 years after harvest)
FI6	Forest harvesting and logging residues removal were conducted using harvester and forwarder in late winter when the area was snow covered. Stump harvest was carried out in early autumn the same year with an excavator. During stump harvest, the ground was not frozen; therefore, deep ruts were formed locally on the harvested areas. Site preparation (mounding) was conducted in the harvested area during summer conditions 1.5 years after the harvest.	During calibration year sampling was conducted 4 times a year. In following years, sampling was conducted monthly during snow free period (1 year before and 4 years after harvest)
FI7	Forest harvesting and logging residues removal were conducted using harvester and forwarder in late winter when the area was snow covered. Stump harvest was carried out in early autumn the same year with an excavator. During stump harvest, the ground was not frozen; therefore, deep ruts were formed locally on the harvested areas. Site preparation (mounding) was conducted in the harvested area during summer conditions 1.5 years after the harvest.	During calibration year sampling was conducted 4 times a year. In following years, sampling was conducted monthly during snow free period (1 year before and 4 years after harvest)

Text S1. Additional information on method for determining stones and boulder content

In each catchment, one harvested area representative of groundwater recharge conditions was selected for determining stones and boulder content using the Viro´s surface penetration method (Viro, 1947). In total, 12 pits were located along two transects at a 90-degree angle to each other, with 6 pits in each line and pits 5 meters apart. Only two people operated the rod and hammer during this study and the force used on the rod was calibrated between the operators. The mean penetration depth from the top of the mineral layer was used as a relative measure of stoniness between the catchments. The depths of the organic layer, to be removed from the total soil penetration depth, were recorded during the soil sampling (for soil analysis) in the recharge area.

Text S2. Additional information on the data treatment

At sites SE2 and SE3, the original study used a BACI design to evaluate effects of stump harvest and site preparation in comparison with stem-harvest only (Eklöf et al., 2013). As we are interested in the harvesting effect in this study, we compared the harvested areas with the non-harvested reference in sites SE2 and SE3. This design was not used in the original publication (Eklöf et al., 2013) as site specific differences between the harvested and non-harvested areas might contribute to concentration differences. However, as we have 23 catchments in the present study, the harvested versus non-harvested comparison was used here for these sites. In the original studies of catchments SE4 and SE5 (Eklöf et al., 2014; Sørensen et al., 2009), treatment effects of logging and site preparation were separated by distinguishing the two-year period between logging and site preparation from the period after site preparation. In this study, we have only included the data from the period after site preparation, representing the combined effect of logging and site preparation measured over 3 years, as site preparation is a common procedure in Swedish forestry. The catchment SE6 study (Eklöf et al., in prep.), separated the effects of logging and stump harvest using a similar approach as for SE4 and SE5, but here we included data representing logging only and excluded data collected after the stump harvest done two years after the logging. In catchment SE1, trees were only removed to create a logging access trail to allow forwarder traffic to pass through the catchment (4% of catchment). Since this was not a regular forest harvest, the SE1 catchment was not included in the statistical evaluation. However, we believe results from this catchment improve our understanding of the variation in forest harvest responses and have therefore included this catchment in tables and

discussion. Kronberg et al. (2016a) studied 10 harvested forest catchments, 5 located below and 5 above the postglacial marine limit (ML), but we only revisited and sampled two of these catchments. Both were located below the ML and mean concentrations of soil C, N, and S, C/N ratio, stone content, disturbance classification and information of harvesting rate and vegetation composition from these two sites were noted for catchment SE7. In the discussion these numbers were related to the overall forestry effect from all sites (n=5) below ML reported in Kronberg et al. (2016a).

Across all study sites, sampling frequency of stream water for MeHg and THg varied from twice a month to monthly (Supplementary material, Table S1). We compared the forest harvesting impacts on runoff MeHg and THg concentrations among these 23 studies. Pre-harvest concentrations were either represented by the mean concentration before harvest or concentrations obtained from unharvested reference catchments. If the original studies reported the size of change as a percent increase (or decrease) after harvest, the post-harvest concentrations were calculated as the pre-harvest concentrations plus the percent increase (or decrease). The concentrations after harvest reported here did not always match the concentration after harvest reported in the original publications, as the size of the treatment effect in the latter may have been calculated with more sophisticated methods than comparing before and after concentrations, e.g., with randomized intervention analysis (RIA; Carpenter et al., 1989). In the current study we have only used concentrations and not loads, as few studies have presented the change in loads of MeHg and THg after harvest. As a result, the forestry effects data presented herein can differ from that in the original study if they were focused on loads and not concentrations (e.g. site SE7 in Kronberg et al., 2016a).

Table S3. The change in THg normalized MeHg concentrations (ΔMeHg/THg) and TOC normalized THg (or DHg) concentrations (ΔTHg/TOC) from before to after harvest. Measured depth of the organic soil horizon, the mean elevation above stream of the harvested area, and the stone and boulder content, measured as mean penetration depth, are included in the OPLS analyses.

**Catchment**	**ΔMeHg/THg**	**ΔTHg/TOC**	**Depth of O horizon (cm)**	**Mean elevation above stream (m)**	**Mean penetration depth (cm)**
US1	*	277	0	51	24
US2	*	267	0	49	13
US3	*	64	0	71	20
US4	*	-100	6	13	22
US5	*	-100	6	8	17
CA1	0,00	***	5	8	17
CA2	**	***	3	27	20
CA3	**	***	4	29	14
NO1	-0,01	76	4	5	18
SE1	0,04	***	10	2	10
SE2	-0,01	-153	9	3	10
SE3	-0,07	-82	3	5	13
SE4	-0,06	47	2	14	22
SE5	-0,05	43	4	1	5
SE6	0,03	41	20	8	27
SE7	0,00	-20	4	1	14
FI1	0,01	***	1	2	20
FI2	-0,05	-64	>100	1	****
FI3	-0,02	-79	>100	1	****
FI4	0,04	-83	>100	1	****
FI5	0,02	-48	>100	0	****
FI6	0,06	-59	>100	0	****
FI7	0,03	-106	>100	1	****

*MeHg below detection limit.
**THg below detection limit.
***No measures of TOC concentrations.
****No reachable mineral layer.

Table S4. Quality assurance and quality control (QA/QC) were determined by using certified reference material.

Reference material	Used for samples from	Certified values of reference material (mg/kg)	Measured average ± standard deviation (mg/kg)
IAEA-456 (Coastal sediment)	Norway, Sweden and Finland	0.077±0.005	0.074±0.001 (n=7)
ERM-CC141 (Loam soil)	Norway, Sweden and Finland	0.083±0.017	0.082±0.006 (n=4)
Mess-3 (Marine sediment)	USA and Canada	0.091± 0.009	0.099±0.004 (n=4)

Table S5. Soil chemistry data, including Hg concentrations, % sulfur, % carbon, % nitrogen and C/N ratio in organic and mineral layer, from the 23 sampling sites. Only the soil chemistry from the organic soil horizon is included in the OPLS analyses. Soil samples were collected during field visits. The table also includes the year forest harvested was conducted.

	Organic soil horizon					Mineral soil horizon					
Catchment	[Hg] mg/kg	%S	%C	%N	C/N ratio	[Hg] mg/kg	%S	%C	%N	C/N ratio	Year of harvest
US1	0,09	0,05	11	0,66	17	0,08	0,04	7	0,31	21	2012
US2	0,13	0,08	23	0,82	27	0,18	0,05	11	0,36	31	2012
US3	0,12	0,11	19	0,99	19	0,12	0,06	10	0,38	28	2012
US4	0,17	0,15	25	1,23	21	0,03	0,02	2	0,12	19	2012
US5	0,12	0,13	15	0,87	16	0,02	0,01	1	0,08	16	2012
CA1	0,23	0,26	37	1,88	20	0,04	0,05	9	0,24	36	2004
CA2	0,15	0,14	25	1,24	19	0,08	0,04	7	0,35	20	2014***
CA3	0,12	0,11	13	0,76	17	0,05	0,03	4	0,21	19	2012-2015***
NO1	0,15	*	44	1,49	29	0,01	*	2	0,05	29	2009
SE1	0,39	*	44	1,59	28	0,06	*	5	0,17	27	1999
SE2	0,30	0,31	39	1,44	27	0,07	0,05	10	0,39	26	2006
SE3	0,15	0,14	28	1,04	27	0,04	0,02	10	0,18	52	2006
SE4	0,16	0,24	36	1,14	31	0,01	0,01	1	0,05	28	2006
SE5	0,01	0,13	42	1,16	36	0,20	0,01	2	0,05	35	2006
SE6	0,18	0,10	15	0,76	20	0,06	0,02	3	0,16	19	2010
SE7	0,25	0,21	41	1,36	30	0,01	0,02	2	0,07	29	2009
FI1	0,13	0,13	15	0,65	23	0,03	0,01	3	0,11	24	1997
FI2	0,32	0,30	49	2,46	20	**	**	**	**	**	2009
FI3	0,15	0,25	54	2,18	25	**	**	**	**	**	2009
FI4	0,10	0,17	52	1,37	38	**	**	**	**	**	2009
FI5	0,07	0,15	54	1,49	36	**	**	**	**	**	2009
FI6	0,42	0,40	51	2,32	22	**	**	**	**	**	2009
FI7	0,21	0,39	51	2,40	21	**	**	**	**	**	2009

*No measures.
**Mineral layer too deep down in ground to allow sampling.
***Harvest date varies within the catchment, harvest within 5 years from sampling started in 2016.

Table S6. The catchment and soil explanatory variables included in the OPLS model describing the change in THg normalized MeHg concentrations (ΔMeHg/THg) in stream waters from before to after harvest ratios (the dependent variable). The coefficients identify the direction of the relationship between the explanatory and dependent variables. The variables with variable influence on projection (VIP)>1 are the most influential in the model and are bolded.

Explanatory variables	Coefficient	VIP
**Depth of organic soil layer**	**0.15**	**1.46**
**Hillslope gradient**	**-0.19**	**1.40**
**%N in organic soil layer**	**0.06**	**1.27**
**Disturbance classification**	**0.11**	**1.11**
**Elevation above stream**	**-0.12**	**1.03**
**Pre-harvest aqueous [THg] or [DHg]**	**0.14**	**1.03**
%S in organic soil layer	0.08	0.99
Pre-harvest aqueous [MeHg]	0.03	0.90
%C in organic soil layer	0.06	0.89
[THg] in organic soil layer	0.08	0.77
C/N ratio in organic soil layer	-0.03	0.65
Harvested proportion (% clear-cut)	0.08	0.52
Mean penetration depth	0.08	0.31

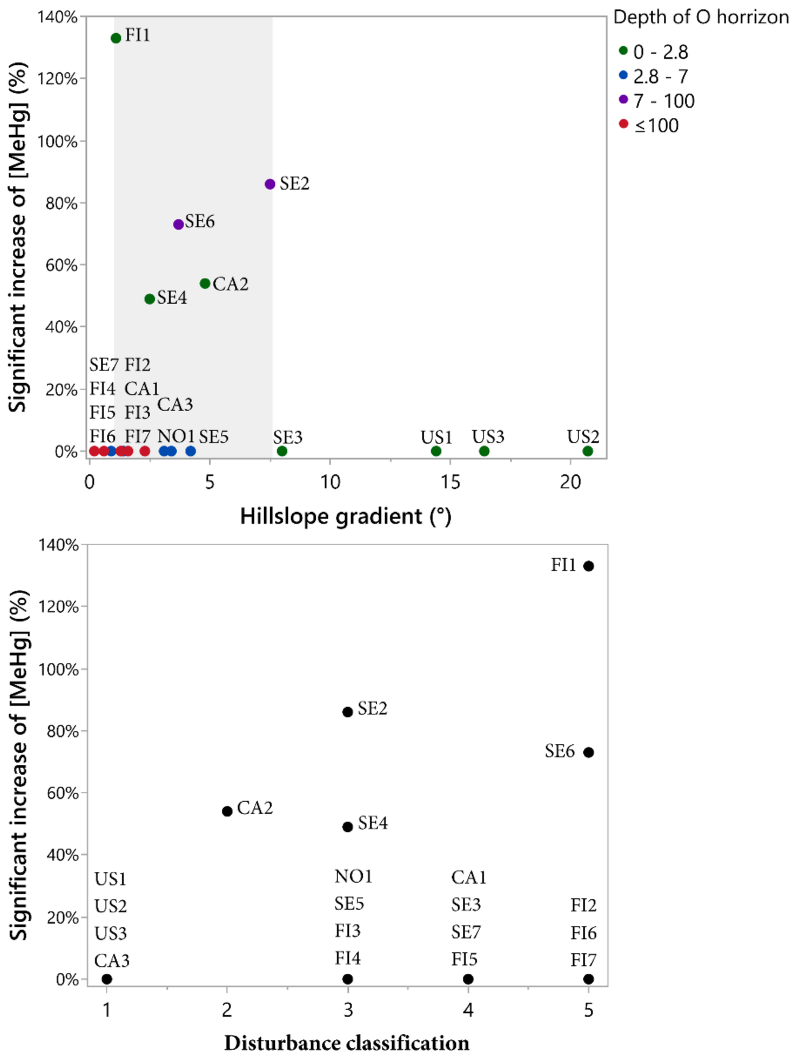

Figure S1. The relationship between the increase of MeHg concentrations from original studies versus hillslope gradient, and soil disturbance classification. The upper figure also show the depth of the organic horizon. The grey area shows the hillslope gradient for the catchments where MeHg in stream waters significantly increased in the original studies. SE1 is not plotted as it did not represent a regular forest harvesting treatment (see Supplementary material, Text S2). Due to limited space, only the country code and a random number, but not the treatment code, are shown in the figure.

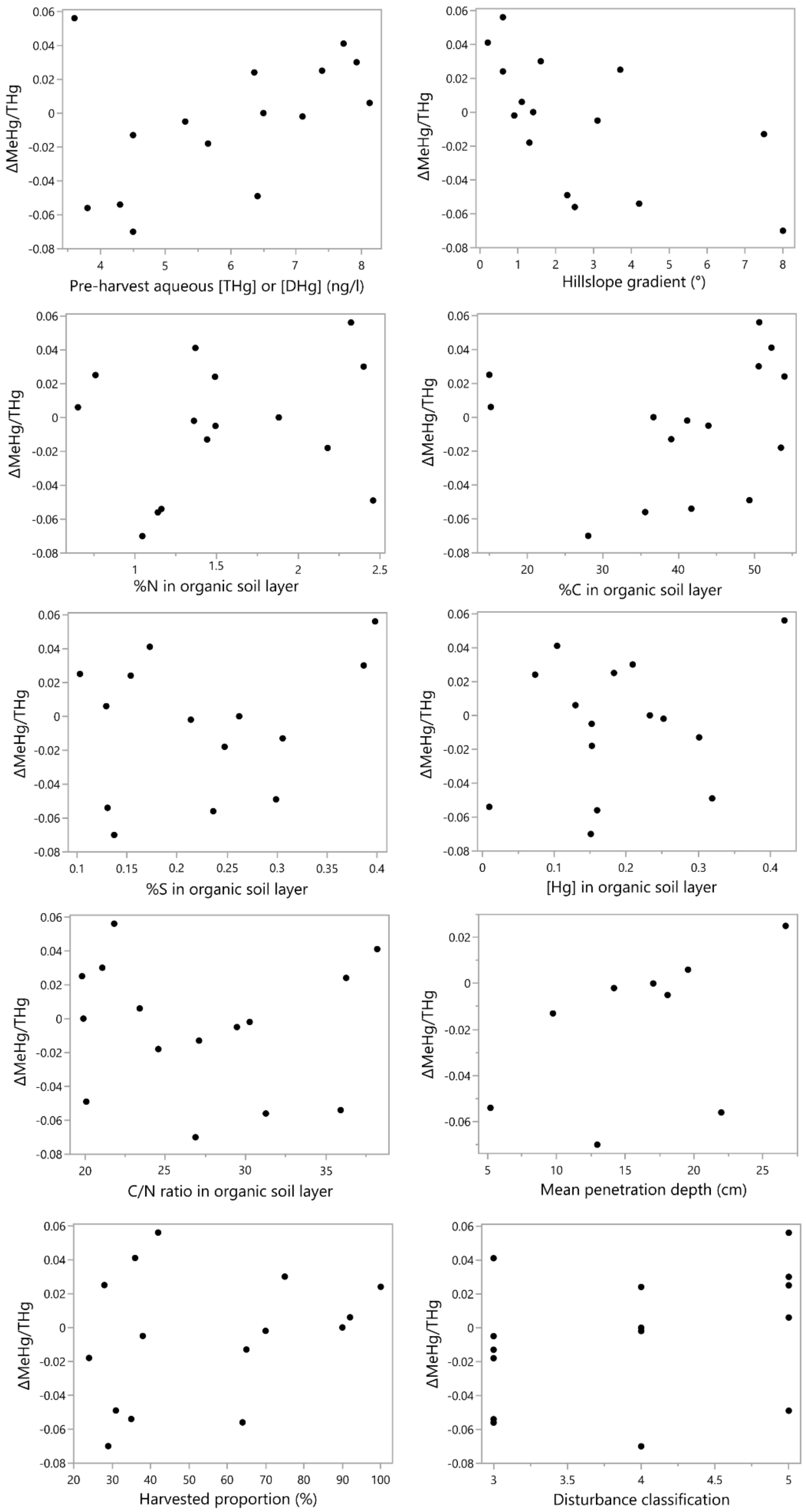
ΔMeHg/THg
Pre-harvest aqueous [THg] or [DHg] (ng/l)
Hillslope gradient (°)
%N in organic soil layer
%C in organic soil layer
%S in organic soil layer
[Hg] in organic soil layer
C/N ratio in organic soil layer
Mean penetration depth (cm)
Harvested proportion (%)
Disturbance classification

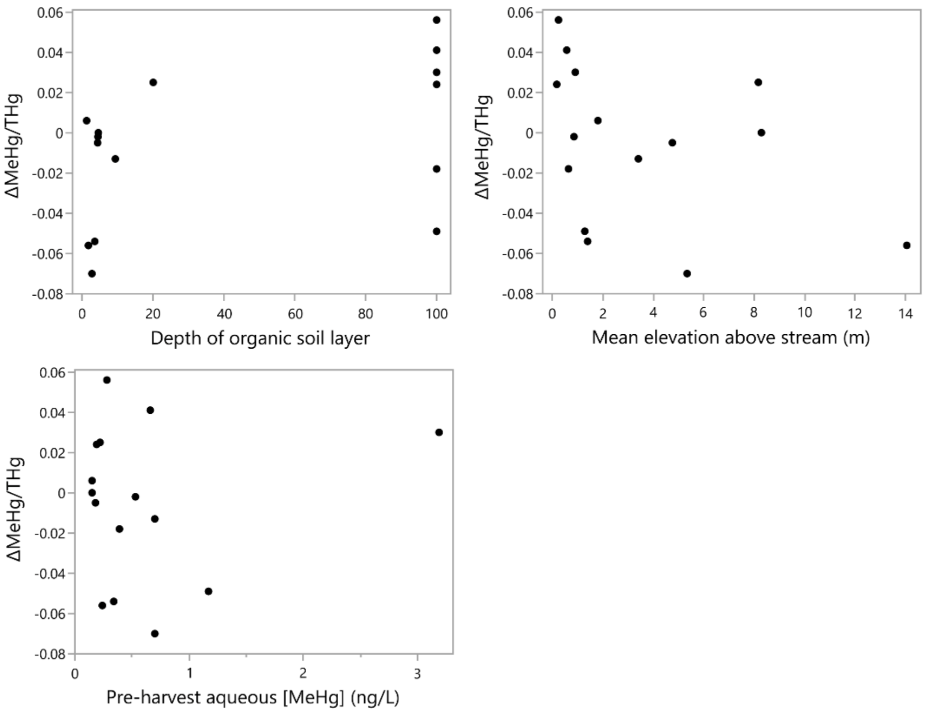

Figure S2. The relationship between the change of MeHg/THg stream water concentrations from original studies versus all explanatory variables included in the OPLS analyses.

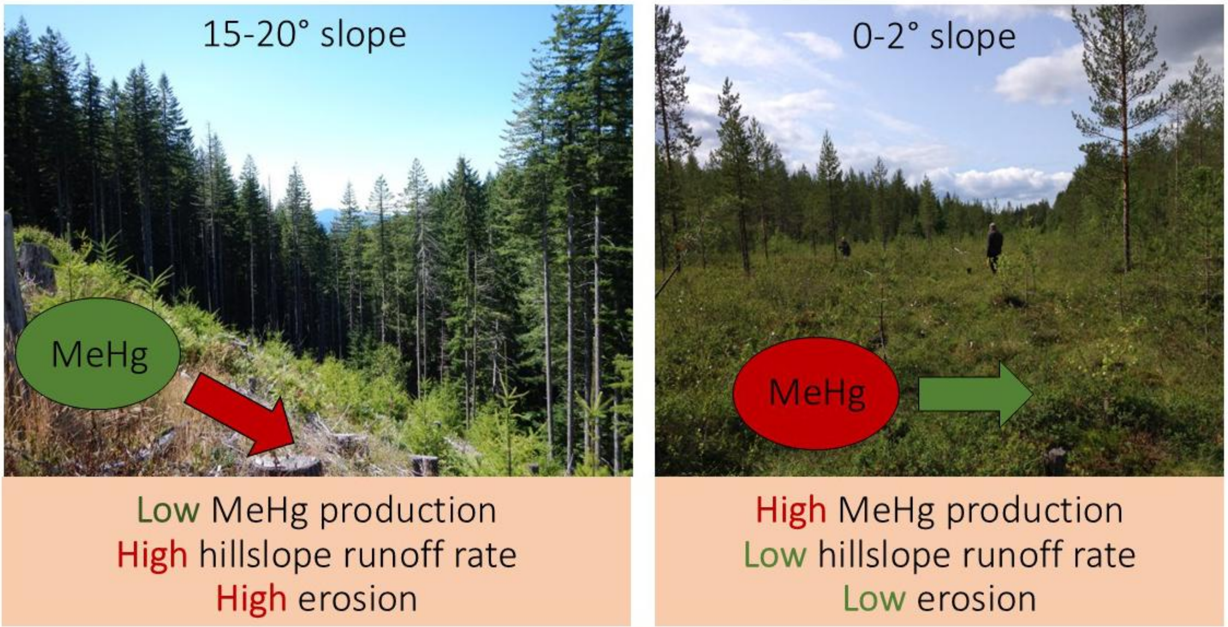

Figure S3. A conceptual figure of the methylmercury (MeHg) formation and mobilization. In the catchments with high hillslope gradients (left) forest harvesting leads to low MeHg formation in soil, but the MeHg hillslope runoff rate that affect water-mediated transport of MeHg to the stream is high. In low hillslope gradients (right) the formation rate of MeHg is high after forest harvest, but due lesser runoff generation, the MeHg mobilization to the streams is low.

Table S7. The explanatory variables included in the OPLS model describing the change in TOC normalized THg concentrations (ΔTHg/TOC) in stream waters from before to after harvest ratios (the dependent variable). The coefficients identify the direction of the relationship between the explanatory and dependent variables. The variables with variable influence on projection (VIP)>1 are most influential in the model and are bolded.

Explanatory variables	M1 coefficient	VIP
**Elevation above stream**	**0.26**	**1.35**
**Hillslope gradient**	**0.11**	**1.19**
**%N in organic soil layer**	**-0.08**	**1.18**
**Pre-harvest aqueous [THg] or [DHg]**	**-0.42**	**1.17**
**%S in organic soil layer**	**-0.14**	**1.13**
**%C in organic soil layer**	**-0.06**	**1.12**
**Disturbance classification**	**-0.02**	**1.03**
**Depth of organic layer**	**-0.06**	**1.00**
[Hg] in organic soil layer	-0.09	0.81
Harvested proportion (% clear-cut)	-0.03	0.67
C/N ratio organic soil	0.05	0.39
Stone and boulders	0.07	0.29

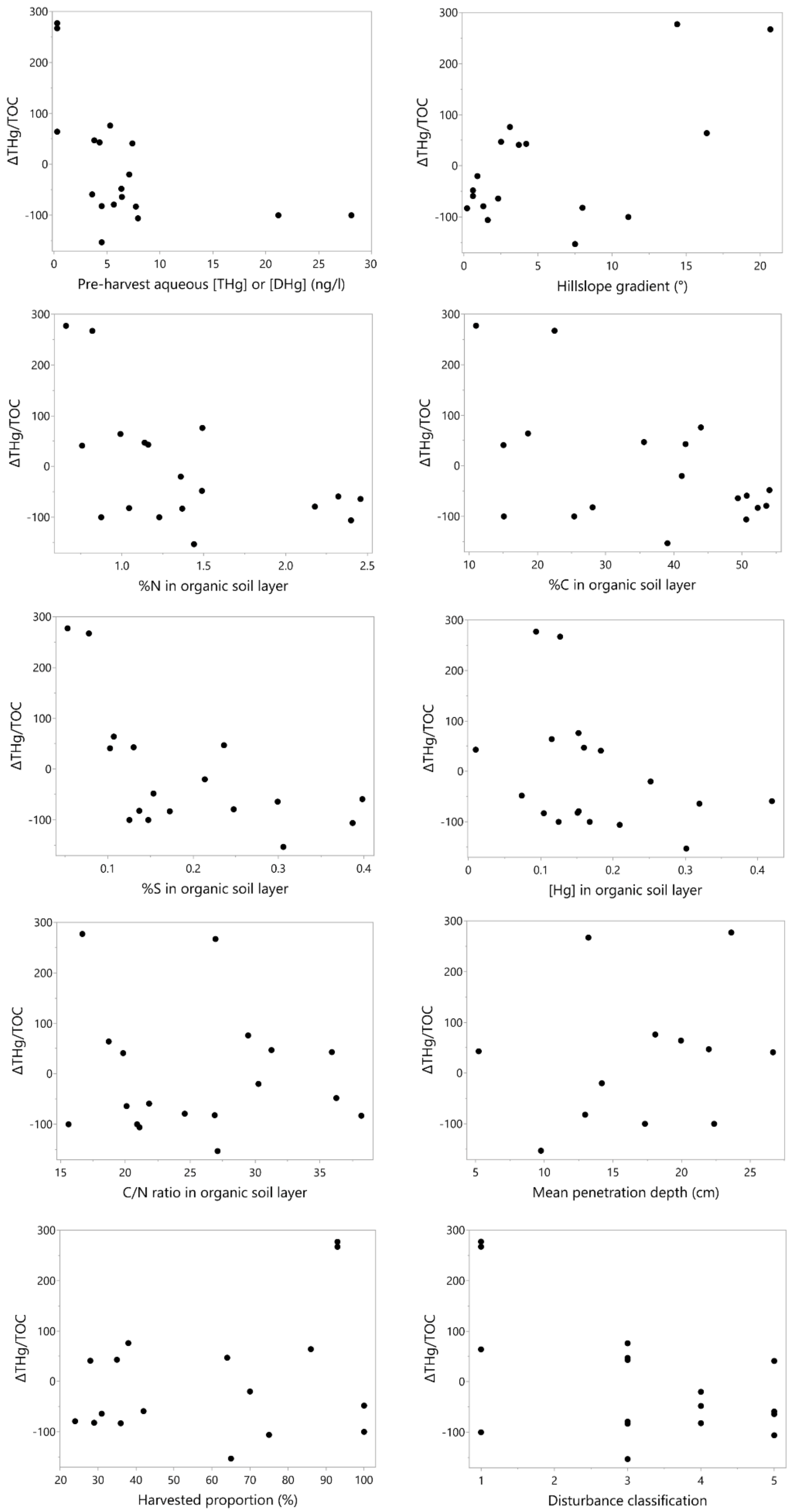
ΔTHg/TOC
Pre-harvest aqueous [THg] or [DHg] (ng/l)
Hillslope gradient (°)
%N in organic soil layer
%C in organic soil layer
%S in organic soil layer
[Hg] in organic soil layer
C/N ratio in organic soil layer
Mean penetration depth (cm)
Harvested proportion (%)
Disturbance classification

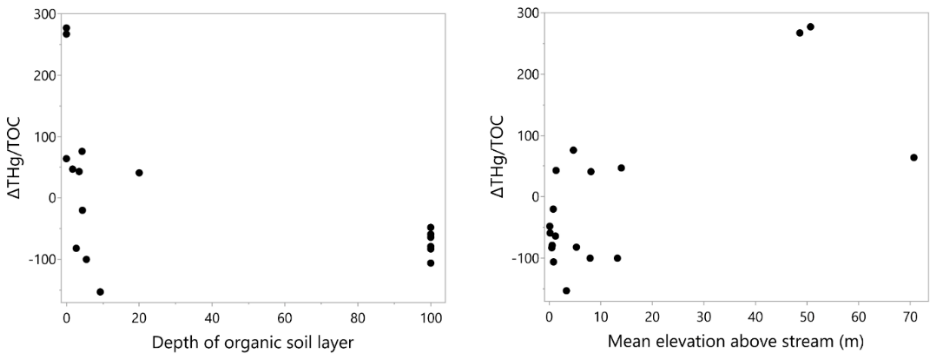

Figure S4. The relationship between the change of THg/TOC stream water concentrations from original studies versus all explanatory variables included in the OPLS analyses.

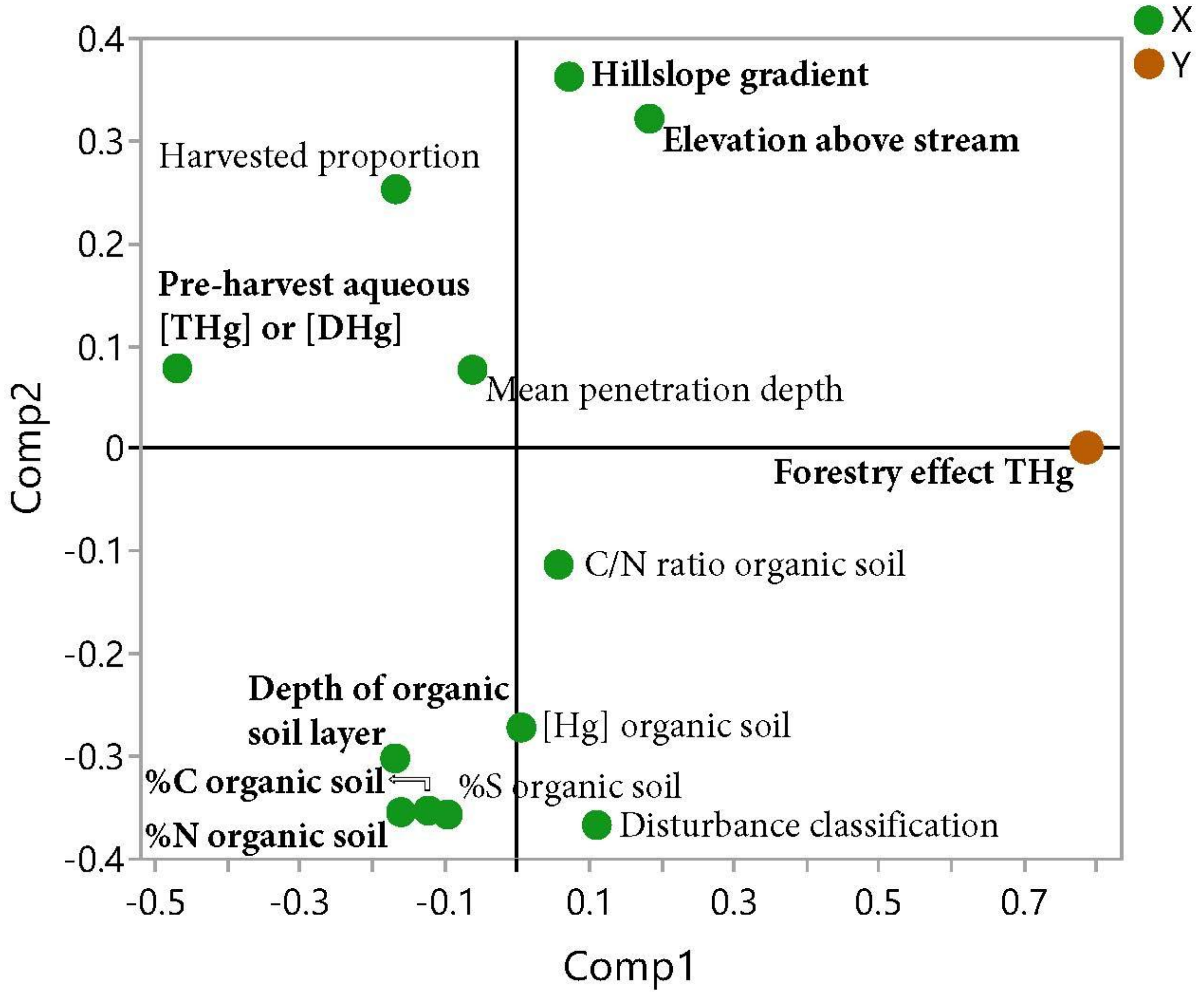

Figure S5. The first two components in the OPLS models explaining the variation across the catchments in the significant change of THg concentrations in stream waters from before (or reference) conditions to after forest harvest. For illustrative purposes we forced the model to include two components, even though the best model included one component. The variables with variable influence on projection (VIP)>1 are most influential in the model and are bolded.

Table S8. The explanatory variables included in the OPLS model with significant increases in THg from before to after harvest ratios as the dependent variable. The coefficients identify the direction of the relationship between the explanatory and dependent variables. The variables with variable influence on projection (VIP)>1 are most influential in the model and are bolded.

Explanatory variables	M1 coefficient	VIP
**Pre-harvest aqueous [THg] or [DHg]**	**-0.34**	**1.42**
**Elevation above stream**	**0.13**	**1.37**
**Hillslope gradient**	**0.07**	**1.08**
**Depth of organic soil layer**	**-0.13**	**1.27**
**%N in organic soil layer**	**-0.16**	**1.37**
**%C in organic soil layer**	**-0.14**	**1.23**
%S in organic soil layer	-0.06	1.15
Disturbance classification	0.12	0.47
Mean penetration depth	-0.04	0.03
% clear-cut in catchment	-0.09	0.01
[Hg] organic layer	0.04	0.61
C/N ratio in organic soil layer	0.03	0.06

References

Allan, C. J., Heyes, A., Mackereth, R. J. 2009. Changes to groundwater and surface water Hg transport flollowing clearcut logging: a Canadian case study. In Does forestry contribute to mercury in Swedish fish? Royal Swedish Academy of Agriculture and Forestry (KSLA) report, 148, 50–54, Stockholm.

de Wit, H. A., Granhus, A., Lindholm, M., M. J., Kainz, Lin, Y., Braaten, H. F. V., Blaszczak, J. 2014. Forest harvest effects on mercury in streams and biota in Norwegian boreal catchments. Forest Ecology and Management 324, 52-63.

Carpenter, S. R., Frost, T. M., Heisey, D., Kratz, T. K. 1989. Randomized intervention analysis and the interpretation of whole-ecosystem experiments. Ecology 70, 1142-1152.

Charbonneau, K. L., Kidd, K. A., Kreutzweiser, D. P., Sibley, P. K., Emilson, E. J. S., O'Driscoll, N. J., Gray, M. A. 2022. Are There Longitudinal Effects of Forest Harvesting on Carbon Quality and Flow and Methylmercury Bioaccumulation in Primary Consumers of Temperate Stream Networks? Environmental Toxicology and Chemistry 41,1490–1507.

Eckley, C. S., Eagles-Smith, C., Tate, M. T., Kowalski, B., Danehy, R., Johnson, S. L., Krabbenhoft, D. P. 2018. Stream Mercury Export in Response to Contemporary Timber Harvesting Methods (Pacific Coastal Mountains, Oregon, USA). Environmental Science & Technology 52, 1971-80.

Eklöf, K., Löfvenius, P., Meili, M., Karlsen, R. H., Bishop, K. Mercury in runoff water as a consequence of forestry: Stem-only harvest versus whole-tree harvest. In preparation.

Eklöf, K., Meili, M., Åkerblom, S., von Brömssen, C., Bishop K. 2013. Impact of stump harvest on run-off concentrations of total mercury and methylmercury. Forest Ecology and Management 290, 83-94.

Kronberg, R-M., Drott, A., Jiskra, M., Wiederhold, J. G., Björn, E., Skyllberg, U. 2016a. Forest harvest contribution to Boreal freshwater methyl mercury load. Global biogeochemical cycles 30, 825-43.

Kronberg, R-M., Jiskra, M., Wiederhold, J. G., Björn, E. Skyllberg, U. 2016b. Methyl Mercury Formation in Hillslope Soils of Boreal Forests: The Role of Forest Harvest and Anaerobic Microbes. Environmental Science & Technology 50, 9177-86.

McCarter, C. P. R., Eggert, S. L., Sebestyen, S. D., Kolka, R. K., Mitchell, C. P. J. 2022. Effects of Clearcutting and Residual Biomass Harvesting on Hillslope Mercury Mobilization and Downgradient Mercury Accumulation. Journal of Geophysical Research: Biogeosciences 127, e2022JG006826.

Munthe, J., Hultberg, H. 2004. Mercury and methylmercury in runoff from a forested catchment - concentrations, fluxes, and their response to manipulations. Water, Air and Soil Pollution: Focus 4, 607-18.

Porvari, P., Verta, M., Munthe, J., Haapanen, M. 2003. Forestry practices increased mercury and methyl mercury output from boreal forst catchments. Environment Science & Technology 37, 2389-93.

Sørensen, R., Meili, M., Lambertsson, L., von Brömssen, C., Bishop, K. 2009. The effect of forest harvest operations on mercury and methylmercury in two boreal streams: Relatively small changes in the first two years prior to site preparation. Ambio 38, 364-72.

Ukonmaanaho, L., Starr, M., Kantola, M., Laurén, A., Piispanen, J., Pietilä, H., Perämäki, P., Merilä, P., Fritze, H., Tuomivirta T. 2016. Impacts of forest harvesting on mobilization of Hg and MeHg in drained peatland forests on black schist or felsic bedrock. Environmental monitoring and assessment 188, 1-22.
